# Synchronization in Multiplex Leaky Integrate-and-Fire Networks With Nonlocal Interactions

**DOI:** 10.3389/fnetp.2022.910862

**Published:** 2022-06-29

**Authors:** K. Anesiadis, A. Provata

**Affiliations:** ^1^ Institute of Nanoscience and Nanotechnology, National Center for Scientific Research “Demokritos”, Athens, Greece; ^2^ School of Applied Mathematical and Physical Sciences, National Technical University of Athens, Athens, Greece

**Keywords:** chimera states, subthreshold oscillations, multilayer networks, excitatory coupling, inhibitory coupling, kuramoto order parameter, correlation function, weak multiplexing

## Abstract

We study synchronization phenomena in a multiplex network composed of two rings with identical Leaky Integrate-and-Fire (LIF) oscillators located on the nodes of the rings. Within each ring the LIF oscillators interact nonlocally, while between rings there are one-to-one inter-ring interactions. This structure is motivated by the observed connectivity between the two hemispheres of the brain: within each hemisphere the various brain regions interact with neighboring regions, while across hemispheres each region interacts, primarily, with the functionally homologous region. We consider both positive (excitatory) and negative (inhibitory) linking. We identify numerically various parameter regimes where the multiplex network develops coexistence of active and subthreshold domains, chimera states, solitary states, full coherence or incoherence. In particular, for weak inter-ring coupling (weak multiplexing) different synchronization patterns on the two rings are supported. These are stable and are obtained when the intra-ring coupling values are near the critical points separating qualitatively distinct synchronization regimes, e.g., between the travelling fronts regime and the chimera state one.

## 1 Introduction

Recent studies in networks of coupled nonlinear oscillators have revealed intriguing synchronization phenomena, which emerge as cooperative effects and can not be predicted by the dynamics of the single oscillators. Common synchronization phenomena in the natural sciences include the rhythmic activity or brainwaves in the central nervous system, the coordinated synchrony in orchestra music, the coordination of simultaneous threads or processes to complete a task (parallel processing) in computer science and, at the opposite end, the dangerous failures of synchrony in telecommunications, in electricity networks and in many other domains of modern technology (Pikovski et al., 2001; Strogatz, 2003; Boccaletti et al., 2018). One notable synchronization example is the partial, local synchronization or chimera state. Chimera states are stable states in oscillator networks, which are composed by coexisting domains of coherent and incoherent oscillators and find various applications, in particular, in the synchronization of neuronal networks (Panaggio and Abrams, 2015; Schöll, 2016; Boccaletti et al., 2018; Omel’Chenko, 2018).

Previous studies of networks composed by Leaky Integrate-and-Fire (LIF) neuronal oscillators with different connectivity schemes have revealed the presence of chimera states with multiple patterns. Luccioli and Politi have studied chimera states in all-to-all coupled networks of nonidentical LIF oscillators (Luccioli and Politi, 2010). Olmi et al., have reported chimera states in two populations of coupled LIF neurons, which contain all-to-all links within each population and one-to-one links between populations (Olmi et al., 2010). Along similar lines, Olmi and Torcini report breathing chimeras and generalized chimera states, where both populations are in partial synchrony, but with different levels of synchronization (Olmi et al., 2019). In the same reference, the authors also study the persistence of chimera states under the influence of disorders, such as random link removal or noise addition to the system. Bolotov et al., also study two populations of all-to-all coupled LIF networks (Bolotov et al., 2016). In their case, the oscillators are identical within each population, but the two populations have different internal frequencies and other parameters. They report phase locking of the mean fields due to the mutual coupling and marginal chimera states in the synchronous population (Bolotov et al., 2016). Rothkegel and Lehnertz identify chimera states in integrate-and-fire populations with small-world connectivity on the torus, considering also refractory period and delays (Rothkegel and Lehnertz, 2014). Considering spatial dimensionality, Tsigkri-DeSmedt et al., have demonstrated chimera states in single (1D) rings, in 2D square (torus) and 3D cubic (hypertorus) lattices of LIF oscillators with nonlocal coupling (Tsigkri-DeSmedt et al., 2016; Schmidt et al., 2017; Tsigkri-DeSmedt et al., 2017; Kasimatis et al., 2018).

In the present study, we complexify the LIF network structure using a multiplex network consisting of two inter-connected rings with nonlocal connectivity within rings and symmetrical one-to-one coupling across rings. We address questions on how synchronization in one ring affects the other, if chimera states are realizable in coupled LIF multiplex networks, what the role of the inter- and intra-coupling strengths is, etc. This multiplex network construction is motivated by recent advances in Magnetic Resonance Imaging (MRI) and in parcellation studies of the human brain. MRI imaging has, up to now, depicted different functional regions and axons bundles at a resolution of the order of 0.1 mm (Finn et al., 2015). In addition, the various human brain parcellation projects have identified a number of functional or structural regions in each hemisphere, with complementary inter-hemispheric connections (Rolls et al., 2015; Arslan et al., 2018; Albers et al., 2021; Chouzouris et al., 2021). In the present multiplex network approach, each hemisphere is roughly represented by one ring (denoted as L-ring for the left ring and R-ring for the right one), while the inter-connections between the L- and R-rings correspond to the neuron axons bundles connecting the left and right hemispheres (Finn et al., 2015). These are severe simplifications with respect to the realistic brain structure and connectivity, however the present study only draws motivation from the brain structure and not exact analogies. All assumptions and simplifications considered in the multiplex network topology and dynamics are discussed in detail in the next sections.

Historically, the domain of partial synchronization and chimera states was initiated by the seminal works of (Kuramoto and Battogtokh, 2002) and by (Abrams and Strogatz, 2004). In both works, the Kuramoto phase oscillator was used to describe the node dynamics. Later on, similar phenomena were reported for other nonlinear oscillators including the FitzHugh-Nagumo oscillator (Omelchenko et al., 2011; Omelchenko et al., 2013; Omelchenko et al., 2015a; Isele et al., 2016; Semenova et al., 2016; Ruzzene et al., 2020; Rybalova et al., 2021; Sawicki et al., 2021), the Hindmarsh-Rose model (Hizanidis et al., 2014), the lattice Limit Cycle model (Hizanidis et al., 2015), the Van der Pol oscillator (Omelchenko et al., 2015b; Omelchenko et al., 2016) and others.

Experimentally, chimera states were reported during the past decade in the domains of mechanics with networks of coupled metronomes (Martens et al., 2013), in catalytic chemical reactions (Tinsley et al., 2012; Nkomo et al., 2016; Kiss, 2018; Totz et al., 2018) and in optical laser lattices (Hagerstrom et al., 2012; Uy et al., 2019). In nature, chimera states have been associated with the uni-hemispheric sleep in sea mammals and migratory birds (Rattenborg et al., 2000; Rattenborg, 2006) and with the settings of epileptic seizures (Mormann et al., 2000; Mormann et al., 2003; Andrzejak et al., 2016).

Most of the works reported above present simulations in networks composed of one ring or of multidimensional tori. Recently, with the expansion of the domain of complex networks the research interest turns toward multiplex networks or even to networks-of-networks. These are networks composed by many layers and are also called multilayer or multidimensional networks. Each layer has independent intra-connectivity and dynamics, but the layers also connect with each other via inter-layer connections (De Domenico et al., 2015; Battiston et al., 2017). Multiplex networks are versatile because they can host many types of interconnected dynamics at different levels. In particular, synchronization phenomena and chimera states on multiplex networks have been reported in the past for the FitzHugh-Nagumo oscillator (Ruzzene et al., 2020; Rybalova et al., 2021; Sawicki et al., 2021) where, by modifying the dynamics or the connectivity in one of the layers, one may influence the activity in the other layers. Similar studies have also been reported for synchronization phenomena in multiplex networks of van der Pol oscillators (Bukh et al., 2020; Shepelev et al., 2021) and the phase and Hindmarsh-Rose models (Maksimenko et al., 2016).

In previous studies on networks of coupled LIF oscillators, one of the present authors (AP) and collaborators have focused on single ring networks with different types of connectivity. Namely, they discussed nonlocal, hierarchical (fractal), reflecting and diagonal connectivities and reported the variations in the synchronization patterns due to structural complexity (Tsigkri-DeSmedt et al., 2016; Tsigkri-DeSmedt et al., 2017; Tsigkri-DeSmedt et al., 2018). Among other findings, they report the emergence of hierarchical chimeras when the connectivity is fractal (Cantor-like), and the coexistence of subthreshold oscillations and chimera states for reflecting connectivity. The present work is in line with these previous studies, focusing on the division of the network in two equivalent subnetworks mimicking the composition of the brain in two hemispheres. In fact, a 2015 study MRI study of the human brain by Finn et al., (Finn et al., 2015), has demonstrated that there are two main types of connectivity between the hemispheres: 1) connectivity between homologous regions (e.g., left parietal lobe to right parietal lobe) and this is called “reflecting connectivity” because the connecting axons link the left with the right hemispheres crossing perpendicularly the plane separating them and 2) scattered/mixed connectivity between various non-homologous brain regions in the two hemispheres (e.g., left parietal lobe with right frontal lobe). In the study by Finn et al., the authors showed that the former connectivity is common to all healthy subjects in the study, while the latter one is unique for each individual and can be used as a person’s MRI fingerprints (see (Finn et al., 2015)). And while the reflecting connectivity accounts for the most basic brain functions common to all, the scattered connectivity accounts for the particular cognitive abilities of each person.

In view of the above, the multiplex connectivity between two layers (rings) can be used as a rough analogue of the reflecting connectivity of the brain, as described in Ref. (Finn et al., 2015). An earlier study used a network structure composed of a single ring divided into two semirings (Tsigkri-DeSmedt et al., 2017). As a result, the connectivity in the junctions between the semirings was mixed, because the nodes near the junctions were linked to both semirings, while nodes away from the junctions were only linked to the opposite semiring. With the present multiplex construction all nodes have common connectivity properties: each node in ring L is linked nonlocally within ring L and has a one-to-one linking with the nodes of ring R and similarly for the nodes of ring R (see Section 2.2).

Using the two-ring multiplex connectivity together with LIF nodal dynamics, we explore numerically the synchrony in the system for excitatory and inhibitory values of inter- and intra-ring connectivities. Various regimes are identified such as coexistence of active and subthreshold domains, chimera states, solitary states, full coherence or incoherence and even different synchronization patterns coexisting on the two rings. In particular, for chimera states the numerical results indicate that the coherent (incoherent) domains are located at identical positions on the two rings when the coupling strengths are high and at opposite positions when the coupling strengths are low. At critical coupling values, which separate the travelling fronts and the chimera domains, it is possible to find simultaneous coexistence of a chimera state in one ring and travelling fronts in the other.

In the next section we present the model, the multiplex coupling scheme and the quantitative synchronization measures to be used in this study. In Section 3 we present the different dynamics regimes when the inter-ring coupling is positive (excitatory interactions). More specifically, active domains mediated by subthreshold elements are recorded when the intra-ring couplings are positive and chimera states emerge when the intra-ring couplings are negative. Similarly, in Section 4 we present the parameter values which support the subthreshold/active coexistence, the travelling fronts and the chimera states, when the inter-ring coupling is negative (inhibitory interactions). In both cases, the Kuramoto order parameter is used as a quantitative measure of synchronization in the network, while the correlation function is used to quantify synchronization between the rings. In Section 5, we discuss the interesting coexistence of travelling fronts in one ring and chimera states in the other, which take place for weak inter-ring coupling strengths. In the Conclusions, we recapitulate our main results and present open problems for future studies.

## 2 The Model

The LIF model describing the dynamics of isolated neurons was first proposed in 1907 by Louis Lapicque (Brunel and van Rossum, 2007; Abbott, 1999). It describes how the isolated neuron reacts under the influence of an external, time-dependent or constant, electrical stimulus.

In the next subsections, first the uncoupled LIF model is briefly recapitulated and then the coupling scheme and the coupled LIF model on multiplex network are introduced. In subsection 2.3 the different measures quantifying the network synchronization are presented.

### 2.1 Brief Recapitulation of the Single LIF Model

Consider the time-dependent membrane potential *u*(*t*) of a nervous cell. Under the influence of an external stimulus *I*(*t*) the membrane potential increases until a specific threshold *u*_th_ is reached. At that time the nervous cell spikes and the potential is reset to its rest state denoted by *u*_rest_. In addition, a leakage term, − *λu*(*t*), is added to the dynamics to avoid divergence of the membrane potential in the absence of resetting. Overall, the dynamics of the single LIF model is described by the following Eq. 1a and condition (1b):

$$\frac{du\left(t\right)}{dt}=\mu -\lambda u\left(t\right)+I\left(t\right)$$ (1a)

$$\underset{\delta t\to 0^{+}}{\lim}u\left(t+\delta t\right)=u_{\text{rest}},when\;u\left(t\right)\geq u_{\text{th}}.$$ (1b)

Eq. 1a represents the integration of the membrane potential, while influx *I*(*t*) may originate from external stimuli or from the collective contributions of the neighboring neurons. Condition (1b) represents the resetting of the potential after reaching the threshold *u*_th_. Namely, the potential *u*(*t*) is reset at *u*_rest_ immediately after (*δt* → 0^+^) its value surpasses the value of *u*_th_. The parameter *μ* in Eq. 1a corresponds to the limiting value of the potential if resetting is not considered. Eq. 1a can be analytically solved, when *I*(*t*) is constant or zero. Then, the constant is incorporated in the parameter *μ* and the solution is: *u*(*t*) = *μ* − (*μ* − *u*_rest_)e^−*t*^, for *u*_rest_ ≤ *u*(*t*) ≤ *u*_th_. The period *T*_*s*_ of oscillations of the single LIF is calculated as $T_{s}=\ln\left[\left(\mu -u_{\text{rest}}\right)/\left(\mu -u_{\text{th}}\right)\right]$ .

Biological neurons are also characterized by a refractory period *T*_*r*_. This is the period of time that the neurons remain at the rest state after resetting. For the sake of simplicity, we will not consider this additional parameter assuming that *T*_*r*_ = 0 and we also set *λ* = 1 owing to time rescaling.

### 2.2 The Coupled LIF Dynamics on Multiplex Connectivity Scheme

**FIGURE 1:**
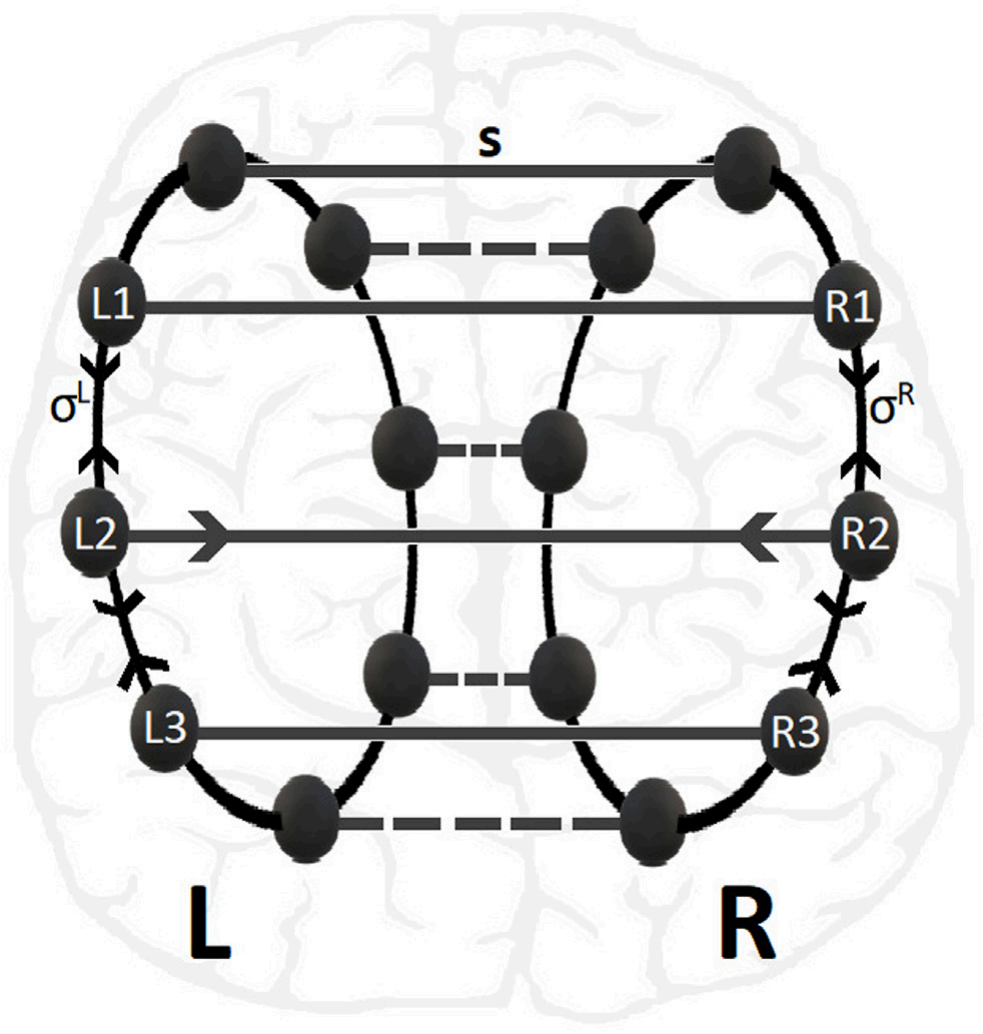
Schematic presentation of multiplex 2-ring connectivity, as motivated by the brain hemispheres structure shown in the background. The nodes in L- and R-rings are linked with inter-ring coupling strength *s* while the nonlocal intra-ring couplings have strengths *σ* ^*R*^ = *σ* ^*L*^. For clarity the connectivity of nodes No. 2 in L- and R-rings are depicted.

Previous studies of the LIF dynamics on a single ring network have demonstrated a variety of synchronization patterns depending on the connectivity (nonlocal, hierarchical, reflecting, small-world, etc), the coupling strength and the coupling range (Luccioli and Politi, 2010; Olmi et al., 2010; Politi and Rosenblum, 2015; Tsigkri-DeSmedt et al., 2016; Tsigkri-DeSmedt et al., 2017; Politi et al., 2018; Tsigkri-DeSmedt et al., 2018; Ullner et al., 2020; Tsigkri-DeSmedt et al., 2021). To keep the system as simple as possible from the point of view of connectivity, in the present study we consider typical nonlocal connectivity within each ring and one-to-one connectivity across rings, see Figure 1.

Let us denote by $\sigma_{ij}^{L}$ the intra-ring connectivity between nodes (*i*, *j*) in ring L and, similarly, for ring R. The links are depicted collectively as *σ* ^*L*^ and *σ* ^*R*^ in Figure 1. For most of the simulations, we assume common values in the intra-ring connectivities to avoid having many different parameters, $\sigma_{ij}^{L}=\sigma_{ij}^{R}=\sigma_{ij}$ . Moreover, when regarding the brain MRI structure, the two hemispheres seem rather symmetrical and we do not have any apriori information on the neuronal connectivity being different in the two hemispheres. Overall, for both rings the general form of the nonlocal intra-ring connectivity with coupling range *K* around node *i* is:

$$\sigma_{ij}^{L}=\sigma_{ij}^{R}=\sigma_{ij}=\left\{\begin{array}{c} \sigma ,\;\forall j:\left[i-K\leq j\leq i+K\right]\;\\ 0,\;elsewhere.\; \end{array}\right.$$ (2)

Only for the calculations of the Kuramoto order parameter in the next two sections we will consider the most general case of different values of *σ* in the two rings and they will be denoted as *σ* ^*L*^ and *σ* ^*R*^, for the left and right rings, respectively.

The inter-ring connectivity between the *i* − th nodes of rings R and L is denoted by $\sigma_{i}^{R\to L}$ and similarly for the opposite direction. Here, there is also no apriori reason to differentiate between R → *L* or L → *R* connectivities and we assume common values for all nodes, $\sigma_{i}^{R\to L}$ = $\sigma_{i}^{L\to R}=s$ . The size of the rings (number of nodes) is denoted by *N* ^*L*^ and *N* ^*R*^ for the left and the right rings, respectively. Let $u_{i}^{L}\left(t\right),\;i=1,\ldots ,N^{L}$ and $u_{i}^{R}\left(t\right),i=1,\ldots ,N^{R}$ represent the membrane potentials of the *i* − th neurons (nodes) in the left and right rings. Then the coupled LIF scheme on the multiplex network reads:

$$\begin{array}{ll} \frac{du_{i}^{L}\left(t\right)}{dt}=\mu -u_{i}^{L}\left(t\right) & +\frac{\sigma^{L}}{2K}\overset{i+K}{\underset{j=i-K}{\sum }}\left[u_{j}^{L}\left(t\right)-u_{i}^{L}\left(t\right)\right]\\ & +s\left[u_{i}^{R}\left(t\right)-u_{i}^{L}\left(t\right)\right] \end{array}$$ (3a)

$$\underset{\delta t\to 0^{+}}{\lim}u_{i}^{L}\left(t+\delta t\right)=u_{\text{rest}},\;when\;u_{i}^{L}\left(t\right)\geq u_{\text{th}}$$ (3b)

$$\begin{array}{ll} \frac{du_{i}^{R}\left(t\right)}{dt}=\mu -u_{i}^{R}\left(t\right) & +\frac{\sigma^{R}}{2K}\overset{i+K}{\underset{j=i-K}{\sum }}\left[u_{j}^{R}\left(t\right)-u_{i}^{R}\left(t\right)\right]\\ & +s\left[u_{i}^{L}\left(t\right)-u_{i}^{R}\left(t\right)\right] \end{array}$$ (3c)

$$\underset{\delta t\to 0^{+}}{\lim}u_{i}^{R}\left(t+\delta t\right)=u_{\text{rest}},\;when\;u_{i}^{R}\left(t\right)\geq u_{\text{th}}.$$ (3d)

In Eq. 3, we consider nonlocal intra-ring connectivity with a coupling range *K*, common in both rings. Note that exchanges between L- and R-rings are possible via the last terms in Eqs. 3a, 3c only, with coupling strength *s*. In the above expressions all the indices in the L-ring (R-ring) are taken mod  *N* ^*L*^ (mod  *N* ^*R*^). Other common parameters of all nodes are the limiting membrane potential value *μ*, the rest state potential *u*_rest_ and the threshold potential *u*_th_.

In this study we use as working parameter set: *μ* = 1, *u*_rest_ = 0, *u*_th_ = 0.98, *K* = 120 and *N* ^*L*^ = *N* ^*R*^ = *N* = 500. For these parameters, the single (uncoupled) rings present chimera states when the coupling strengths take negative values and subthreshold oscillations for positive ones. The *σ* − values in the multiplex connectivity vary in the range −2 ≤ *σ*_*ij*_ ≤ 2. All simulations start from random initial conditions, while periodic boundary conditions are considered for all indices.

### 2.3 Synchronization Measures

For quantifying the synchronization within each ring two measures are employed here, the mean phase velocity *ω*_*i*_ (frequency of oscillations) of node *i* and the Kuramoto order parameter *Z*. The mean phase velocity which counts the number of complete cycles *Q*_*i*_ performed by oscillator *i* during the time interval Δ*T* divided by Δ*T* is used for quantifying the frequency difference between nodes (Omel’Chenko, 2018; Omelchenko et al., 2013; Omelchenko et al., 2015a). It is defined as:

$$\omega_{i}=\frac{2\pi Q_{i}}{\Delta T}.$$ (4)

The time interval Δ*T* in the present study is chosen between 300 and 500 cycles, depending on the parameter values. The terms mean phase velocity *ω*_*i*_ and frequency *f*_*i*_ of oscillator *i* are proportional, *ω*_*i*_ = 2*πf*_*i*_, and thus they will be used interchangeably in the text.

The Kuramoto order parameter *Z* is a synchronization measure for quantifying synchrony in an ensemble of oscillators (Omel’Chenko, 2018; Kuramoto and Battogtokh, 2002). In this case, it will be used for quantifying synchrony within a single ring. For defining *Z* ^*L*^ (Kuramoto index in L-ring) we first need to define the phase $\phi_{i}^{L}$ of oscillator *i* in ring L. For the LIF oscillator the instantaneous phase $\phi_{i}^{L}\left(t\right)$ is defined as (Argyropoulos and Provata, 2019):

$$\phi_{i}^{L}\left(t\right)=\frac{2\pi u_{i}^{L}\left(t\right)}{u_{\text{th}}}.$$ (5)

Similarly, are defined the phases in the ring R. Then, the instantaneous Kuramoto order parameter which defines synchronization in ring L is defined as:

$$Z^{L}\left(t\right)=\frac{1}{N^{L}}\left|\overset{N^{L}}{\underset{k=1}{\sum }}e^{i\phi_{k}^{L}\left(t\right)}\right|,$$ (6)

where |⋅| stands for the magnitude of the complex number in the argument and the sum runs over the number of elements, *N* ^*L*^. Similarly, the Kuramoto order parameter *Z* ^*R*^(*t*) is defined for ring R. In general, the order parameter takes values in the range 0 ≤ *Z*(*t*) ≤ 1. When *Z* ≃ 0 then the ring elements are asynchronous (full disorder) and when *Z* ≃ 1 they are synchronous (full synchrony). Intermediate values of *Z* indicate partial network synchronization (chimera state).

To quantify synchronization between the two rings the Kuramoto order parameter can be considered over the entire multiplex as:

$$Z^{L-R}\left(t\right)=\frac{1}{N^{L}+N^{R}}\left|\overset{N^{L}}{\underset{k=1}{\sum }}e^{i\phi_{k}^{L}\left(t\right)}+\overset{N^{R}}{\underset{k=1}{\sum }}e^{i\phi_{k}^{R}\left(t\right)}\right|.$$ (7)

This is different from the difference of the two Kuramoto order parameters $\left|Z^{L}-Z^{R}\right|$ (see next two sections).

An alternative measure of inter-synchrony between rings R and L is the linear Pearson’s correlation function *C* ^*L*−*R*^, defined as (Nicosia and Latora, 2015):

$$C^{L-R}\left(t\right)=\frac{\left\langle u_{i}^{L}u_{i}^{R}\right\rangle -\left\langle u_{i}^{L}\right\rangle \left\langle u_{i}^{R}\right\rangle }{\sqrt{\left(\left\langle u_{i}^{L}u_{i}^{L}\right\rangle -{\left\langle u_{i}^{L}\right\rangle }^{2}\right)\left(\left\langle u_{i}^{R}u_{i}^{R}\right\rangle -{\left\langle u_{i}^{R}\right\rangle }^{2}\right)}},$$ (8)

where the averages $\left\langle \cdot \right\rangle$ are defined over all *i* = 1, … , *N* elements in each ring. The numerator in expression (8) represents the covariance between L- and R-rings and the denominator is the product of the variances of the two rings. For full synchronization between the two rings the magnitude of the correlation function $\left|C^{L-R}\right|\to 1$ .

Regarding negative coupling strengths, where subthreshold together with oscillatory domains are found, appropriate measures are the activity factors, *A* ^*L*^ and *A* ^*R*^, which count the average number of elements that escape the threshold and perform complete cycles for the L- and R-ring. In particular, for the L-ring the average activity factor is defined as:

$$A^{L}=\frac{1}{N^{L}\Delta T}\overset{\Delta T}{\underset{t=0}{\sum }}\overset{N^{L}}{\underset{i=1}{\sum }}H\left(u_{\text{th}}-u_{i}^{L}\left(t\right)-\epsilon \right).$$ (9)

Here, *H*(*n*) is the Heaviside function defined as *H*(*n*) = 0 for *n* < 0 and *H*(*n*) = 1 if *n* ≥ 0 and *ϵ* is a tolerance factor that excludes the counting of the subthreshold elements, facilitating the calculations. In the present study, the tolerance value *ϵ* = 0.01 is used. To avoid temporal fluctuations the ring activities are also averaged over a time period Δ*T*, after the system has reached the steady state. Normally, Δ*T* comprises of 300–500 cycles depending on the parameter values. Similarly, the activity factor for the R-ring is defined.

The mean phase velocity distribution, the Kuramoto order parameters, the correlation function, the system activities and the other quantitative measures depend on the model parameters (intra- and inter-ring coupling strengths in the present case).

## 3 Synchronization Patterns for Positive Inter-Ring Coupling

In computational neuroscience, both positive and negative coupling strengths are considered, drawing from experimental findings about excitatory (approx. 70%) and inhibitory (approx. 30%) coupling between brain neurons. In the present section, the excitatory (positive) coupling is studied for the inter-ring connectivity between the two rings, while the intra-ring connectivity may take positive and negative values.

For the numerical studies, the number of nodes was chosen to be *N* ^*L*^ = *N* ^*R*^ = 500 in each ring. This choice seems reasonable for two main reasons.

1 The various human brain parcellation studies, which divide the brain in structural or functional centers, have used a number of brain parcels (nodes) varying from 70 to 360 (Rolls et al., 2015; Arslan et al., 2018; Albers et al., 2021; Chouzouris et al., 2021). Therefore, a number of nodes of the order of 500 for each ring covers adequately the computational brain division.
2 From previous studies of the LIF and FitzHugh Nagumo networks we have seen that a number of 500–1000 nodes is good enough to avoid finite size effects which are dominant in smaller sizes (Tsigkri-DeSmedt et al., 2020).

**FIGURE 2:**
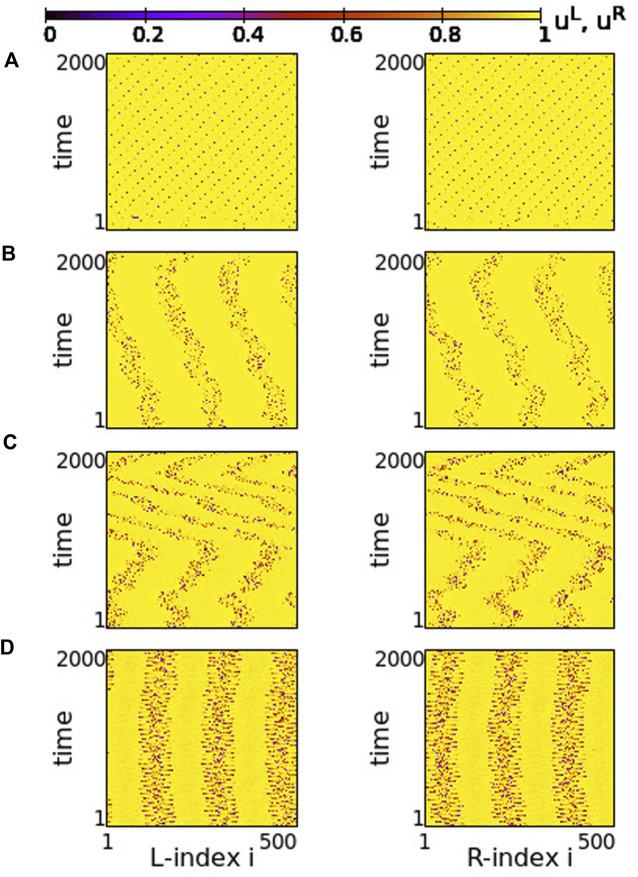
Spacetime plots of the potentials for the L- and R-rings on the left and right panels, respectively. **(A)** *σ* = +1.9, **(B)** *σ* = +1.2, **(C)** *σ* = +0.7 and **(D)** *σ* = +0.4. Other parameters are: *N* ^*L*^ = *N* ^*R*^ = *N* = 500, s = +0.1, *K* = 120, *μ* = 1, *u*_rest_ = 0, *u*_th_ = 0.98. All simulations start from the same random initial conditions.

**FIGURE 3:**
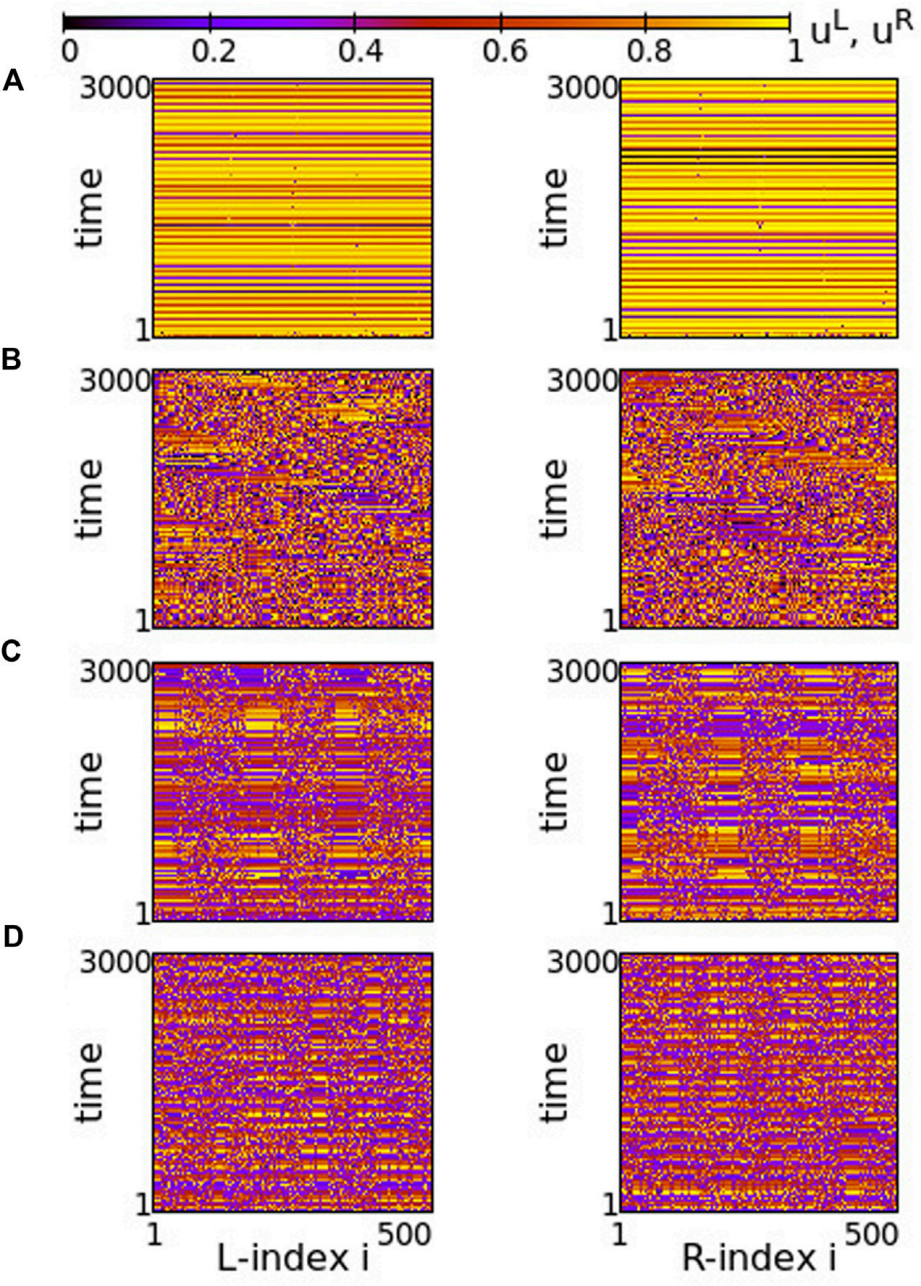
Spacetime plots of the potentials for the L- and R-rings on the left and right panels, respectively. **(A)** *σ* = −0.2, **(B)** *σ* = −0.9, **(C)** *σ* = −1.7 and **(D)** *σ* = −2.0. Parameter *s* = +0.1 and other parameters are as in Figure 2.

Using the above system size and the working parameter set described at the end of Section 2.2, we performed numerical simulations for different values of the coupling strengths *σ* and *s*. Typical synchronization patterns are presented in Figure 2 for *σ* > 0 and in Figure 3 for *σ* < 0.

In Figure 2, for *σ* > 0 and weak coupling *s* = 0.1 between the two rings, we show the spacetime plots of the potentials to demonstrate coherence between the two rings; left panels correspond to L-ring and right panels to R-ring. In both rings, we note regions where the elements do not perform full oscillations but remain subthreshold. These (yellow-coloured) regions are separated by active regions. For large positive values, *σ* > 1.7, most of the elements remain subthreshold, while isolated elements perform full oscillations, see Figure 2A. Note that there is an overall motion to the left for both rings. For intermediate coupling ranges, 0.7 < *σ* < 1.6, the system separates into six distinct regions, which alternate between active and subthreshold domains. The active regions move erratically to the left and to the right. Regarding their positions on the two rings, the active regions are located at the same positions and their erratic motion has the same tendency (is in the same direction) in both rings, see Figures 2B,C. This is a result of the coupling *s* between the two rings which causes coherence. Note that, in Figure 2A, for short times the rings also attempt to create three active regions alternating with subthreshold regions, for times *t* < 200 time units (TUs). For these large coupling strengths, the division fails and we soon have the creation of single oscillating elements with transportation of the activity around the ring.

For small positive coupling strengths, the active regions stabilize in space in both rings, see Figure 2D. At the same time, we note that the position of the active (subthreshold) regions in one ring is covered by subthreshold (active) regions in the other ring. In other words, at the positions where in the L-ring we encounter active regions, in the R-ring these regions are subthreshold. This is counter-intuitive since the elements are in a one-to-one correspondence in the two rings and are directly coupled with strength *s*. This behaviour is encountered for 0.2 ≤ *σ* ≤ 0.4, while for *σ* → 0, the motion in each ring becomes incoherent. However, coherence is achieved between elements at equivalent positions on the two rings due to the non-vanishing inter-ring coupling *s* (see Supplementary Figure S3).

The division of the network in coexisting domains of active and subthreshold elements is not due to the multiplex structure of the network. It has been observed earlier in single ring dynamics, for positive coupling strengths (Tsigkri-DeSmedt et al., 2017). What is new and unexpected in the multiplex connectivity is the establishment of domains with different activity in connected regions of the two rings, as in Figure 2D.

In all above cases we have considered a constant coupling range, *K* = 120, in the system. In single ring networks the coupling range defines the size of the active/subthreshold or coherent/incoherent regions (Tsigkri-DeSmedt et al., 2017). The same holds true here. For larger coupling ranges fewer active/subthreshold domains are created (see Supplementary Figure S4).

As a final comment for the case *σ* > 0, we recall that if the resetting condition is not considered the single elements have a fixed point *u*_fixed_ = *μ* = 1. When the coupling is introduced, the fixed point can be displaced. When the displacement causes that *u*_fixed_ reaches values below *u*_th_ (recall that *u*_th_ = 0.98 in the present study) then some elements are attracted by this fixed point and create domains of subthreshold elements as in Figure 2.

Regarding the negative values of the coupling strengths, the numerical results show that the subthreshold elements disappear and all elements perform full oscillations. In Figure 3, typical spacetime plots of the potentials are shown for different values of *σ*, keeping *s* = +0.1. For small negative values of the intra-ring coupling strength, − 0.6 ≤ *σ* < 0, solitary states are formed, see Figure 3A. These are isolated oscillators that deviate from an otherwise coherent background. Note that they are formed at identical positions in the two rings. As the modulus of *σ* increases (*σ* becomes “more negative”), the isolated solitaries tend to mobilize creating incoherent regions of increasing sizes (see Supplementary Figure S5). This way, the isolated solitaries give rise to typical chimera states. An attempt of the system to create such a chimera state is shown in Figure 3B, where coherent and incoherent domains appear randomly in the two rings. As the modulus of *σ* increases further, typical chimera states emerge with coherent and incoherent elements located at identical positions on the two rings, see Figure 3C. For even higher moduli of negative coupling strengths, shooting solitaries appear within the coherent regions in the two rings and destabilize them (Figure 3D), driving the system to full incoherence.

**FIGURE 4:**
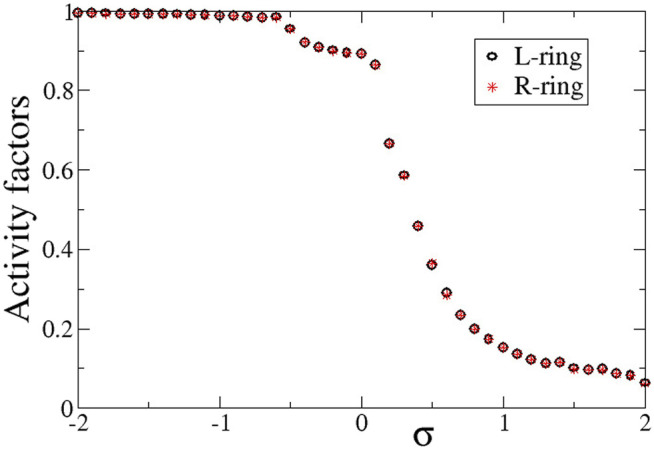
The average activity factors, *A* ^*L*^ (circles), and *A* ^*R*^ (stars) versus the intra-ring coupling strength *σ*. Temporal averages are taken over Δ*T* = 800 TUs, after excluding the first 200 TUs as transients. Parameter *s* = +0.1 and other parameters are as in Figure 2.

Quantitatively, the synchronization properties of the multiplex network are evaluated via the Kuramoto order parameter, the activity factors and the correlation function, as described in Section 2.3. In Figure 4 we present the average activity factors *A* ^*L*^ (black open circles) and *A* ^*R*^ (red stars) in the L- and R-ring, respectively (see Eq. 9). For the activity calculations temporal averages were taken over Δ*T* = 800 TUs after the system has reached the steady state. In corroboration with Figures 2, 3, for positive values of *σ* ^*L*^ = *σ* ^*R*^ = *σ* a number of elements stay subthreshold (do not oscillate) and therefore, the activity factors are less than unity. Our simulations show that the number of elements that oscillate decreases with *σ* (while the rest remain subthreshold). On the contrary, for negative values of *σ*, all elements oscillate, independently of whether they belong to the coherent or the incoherent part of the chimera state. Therefore, for *σ* < 0, *A* ^*L*^ ∼ *A* ^*R*^ ∼ 1. We note that there can be a deviation from unity in the case of *σ* < 0. This is attributed to the counting of the subthreshold elements. In many cases, active elements that perform full oscillations can be momentarily found in the region $\left[u_{\text{th}}-\epsilon ,u_{\text{th}}\right]$ and in this case they are mistakenly counted as subthreshold elements, underestimating consistently the percentage of active oscillators. We also note that the activity is almost identical in both rings, as the coupling strength is the same in both, *σ* ^*L*^ = *σ* ^*R*^ = *σ*.

**FIGURE 5:**
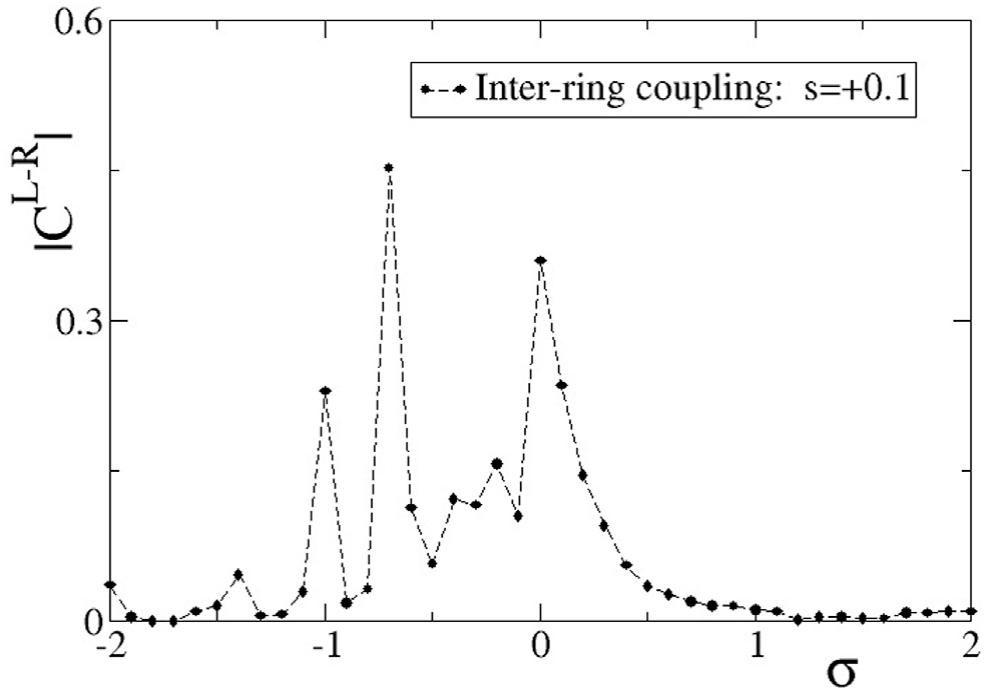
The correlation function, $\left|C^{L-R}\right|$ , versus the intra-ring coupling strength *σ*. Parameter *s* = +0.1 and other parameters are as in Figure 2.

In Figure 5, we present the absolute value of the correlation function, $\left|C^{L-R}\right|$ , versus the intra-ring coupling strength *σ*, with inter-ring coupling strength *s* = +0.1. Other parameters are set to the working parameter set. For the calculation of $\left|C^{L-R}\right|$ in Figure 5, except for the average over the elements on the L- and R-ring, a time average is also considered to account for the fluctuations during the simulation. For the time average in the calculations of the correlations the first 1000 TUs were disregarded (considered as transient) and the average was taken over the subsequent 2000 TUs as a function of the intra-ring coupling strength *σ*.

For positive, large values of *σ*, $\left|C^{L-R}\right|$ takes small values indicating absence of correlations between the L- and R-ring. Inspecting closely Figure 2A, the travelling waves show identical traits in the two rings, but their positions are in different locations on the rings. In addition, the subthreshold elements are located at random positions below the threshold, uncorrelated between the L- and R-ring. That is why the correlation function drops to zero. As the value of *σ* decreases, keeping positive values, the subthreshold and active regions acquire a certain degree of common location in the two rings and this causes the $\left|C^{L-R}\right|$ function to increase. This is more evident for values of, e.g., *σ* = 0.2 (see Supplementary Figure S6). Maximum value is attained for intra-ring coupling equal to zero, where only inter-ring coupling is present and causes a relatively high degree of correlation between L- and R-ring.

As the *σ* values decrease entering negative values in Figure 5, we note a certain decrease in the L-R correlations, although the two rings are both almost synchronous, see Figure 3A. The small inter-ring correlation is due to a phase difference that is established between the two rings, due to the different initial conditions. The small values of the inter-ring coupling are not sufficient to enforce full synchrony between the two rings, which operate with a certain constant phase gap. This behaviour dominates for −0.8 < *σ* < 0. For smaller values of *σ* < − 0.8, chimera states are formed, as also Figures 3B–D demonstrate. The correlation function drops toward 0, since in the incoherent domains the phases are disordered in the two rings and also the coherent domains often have a phase gap keeping the correlation values low.

**FIGURE 6:**
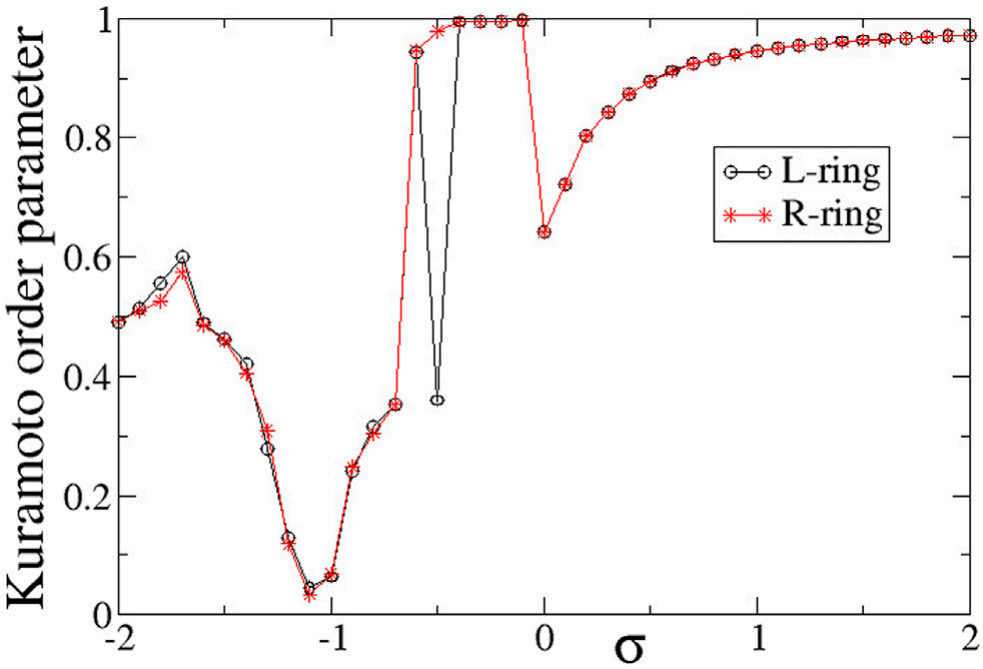
The Kuramoto order parameters, *Z* ^*L*^ (circles) and *Z* ^*R*^ (stars), versus the intra-ring coupling strength *σ* for positive inter-ring coupling *s* and equal intra-ring couplings *σ* ^*L*^ = *σ* ^*R*^ = *σ*. Parameter *s* = +0.1 and other parameters are as in Figure 2.

To quantify synchronization in the rings, the Kuramoto order parameters are calculated and plotted in Figure 6. For intra-ring couplings *σ* < − 0.6, where typical chimera states are formed, both *Z* values are distinctly lower than 1, in accordance with the chimera presence. In the small magnitude of the negative couplings, 0.6 < *σ* < 0, where we note the presence of solitaries, the Kuramoto order parameters tend to 1, indicating almost full coherence. This is not unexpected, since the solitaries are rare and only occasionally disturb full coherence, recall Figure 3A. For positive *σ* values with relatively small magnitudes, 0 < *σ* ≤ 1.0, again the *Z* values are small, indicating incoherence in the system. We recall that in this *σ*-range the system presents alternations of subthreshold (homogeneous) regions and active (incoherent) domains. As *σ* increases the width of the active, incoherent regions decreases. This behaviour continues above *σ* > 1.0, where the incoherent regions are as small as only isolated elements. This leads to *Z* → 1, as *σ* increases.

In Figure 6, it is interesting to note that the behaviour is very similar in the two rings, except for the case *σ* = −0.5, where *Z* ^*L*^ = 0.36 and *Z* ^*R*^ = 0.98. This discrepancy takes place precisely at the parameter *σ* values where there is a transition between chimera states (*Z* < 1) and solitary states (*Z* → 1) and it is a typical signature of qualitative change of behaviour in dynamical systems.

**FIGURE 7:**
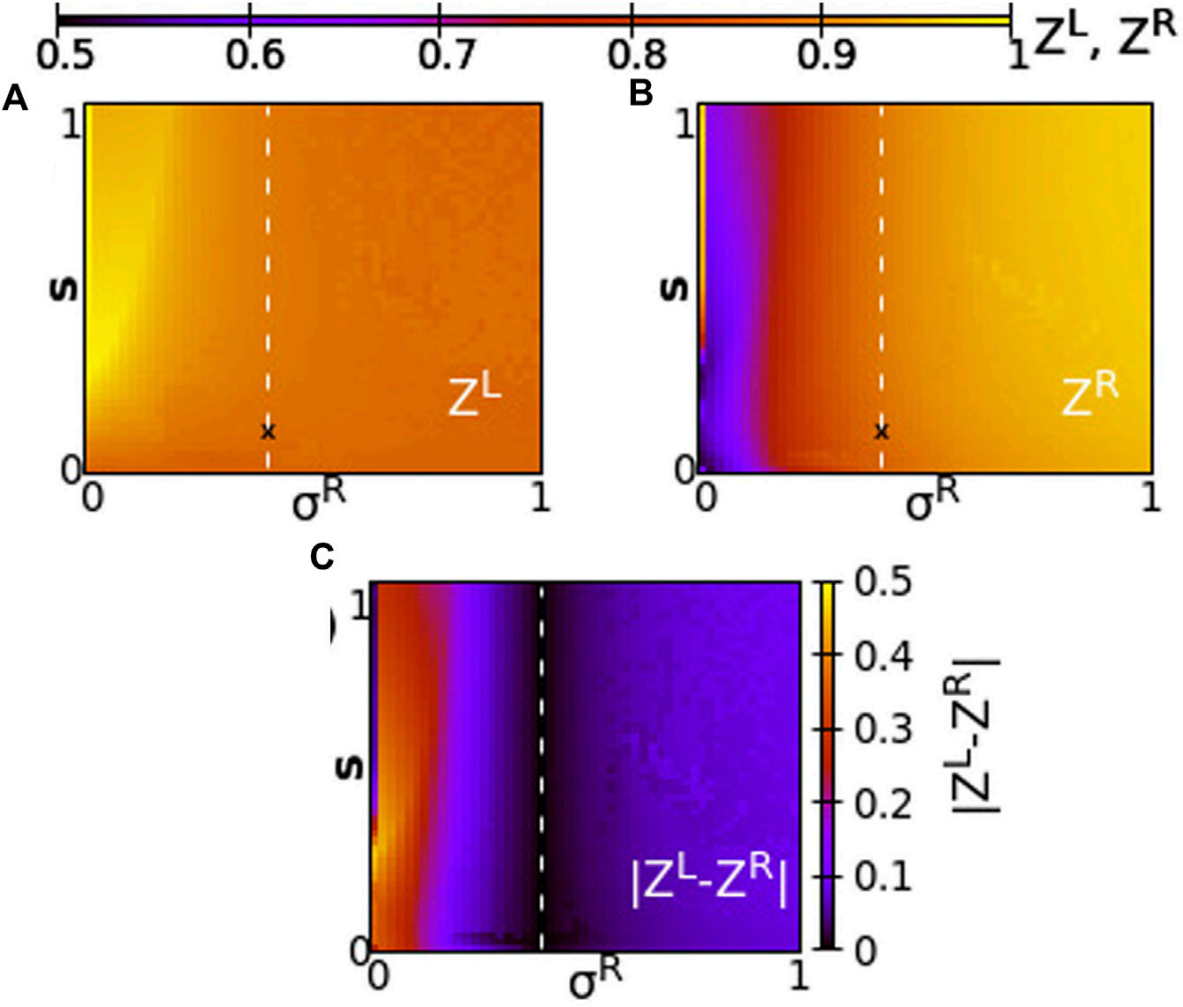
The Kuramoto order parameters *Z* ^*L*^ and *Z* ^*R*^ represented on the color scale for different values of the intra-ring coupling constant in ring R, *σ* ^*R*^, and the inter-ring coupling constant, *s*. **(A)** *Z* ^*L*^, **(B)** *Z* ^*R*^ and **(C)** $\left|Z^{L}-Z^{R}\right|$ . The white dashed lines represent equal intra-ring couplings *σ* ^*L*^ = *σ* ^*R*^ = 0.4 and variable $s\in \left[0,1\right]$ . The “X” symbol marks coupling strengths *σ* ^*L*^ = *σ* ^*R*^ = 0.4 and *s* = 0.1, discussed in Figure 2D. Parameter *σ* ^*L*^ = +0.4 and other parameters are as in Figure 2.

Taking a different perspective, we now consider the synchrony in the system when the two rings have different coupling strengths, *σ* ^*L*^ ≠ *σ* ^*R*^. We, then, address questions such as, When do the rings synchronize? Does one ring lead the other? Are the synchrony patterns symmetric in both rings? To answer these questions, we perform numerical simulations of the network keeping constant *σ* ^*L*^ = 0.4 and we vary *σ* ^*R*^ in the range [0,1] and inter-ring coupling *s* in the range [0,1]. This way all couplings are positive in the system. Other parameters are taken as in the working parameter set. To quantify the synchrony in the system, we compute the Kuramoto order parameters *Z* ^*L*^ and *Z* ^*R*^, and the absolute value of the difference $\left|Z^{L}-Z^{R}\right|$ , as discussed in Section 2.3. The last one accounts for the difference in synchronization between the two rings. In the plots of Figure 7, the values of the Kuramoto order parameter are represented by the color scale. The coupling strength *σ* ^*R*^ varies along the *x*-axis and the inter-ring coupling *s* varies along the *y*-axis. *σ* ^*L*^ = 0.4 remains constant in all simulations. The “X” mark in the three panels corresponds to the position *σ* ^*R*^ = 0.4, s = 0.1, for which *σ* ^*L*^ = *σ* ^*R*^ = 0.4 and corresponds to Figure 2D.

Since all coupling strengths are positive, only subthreshold oscillations and coexisting active regions are recorded in the system, similar to Figure 2. Chimera states are not observed when *σ* ^*L*^, *σ* ^*R*^, *s* > 0. We first note that synchronization in both rings takes similar values when *σ* ^*L*^ ∼ *σ* ^*R*^, around the position *σ* ^*R*^ = *σ* ^*L*^ = 0.4 and for all values of *s* (see dashed line). To the left of the dashed line, we note moderate synchronization in the left ring since the intra-ring coupling strength remains to moderate values, *σ* ^*L*^ = 0.4, while in the right ring the synchronization is facilitated, as *σ* ^*R*^ increases toward 1. To the left of the dashed line, for small values of *σ* ^*R*^, the synchrony is high on the L-ring (recall that *σ* ^*L*^ = 0.4 is always kept constant) and is low in the R-ring, where the small values of *σ* ^*R*^ prevent high synchronization. It is remarkable that the value of *s* plays a minor role in the degree of synchronization, except in the cases of fairly small *σ* ^*R*^ values. Regarding the values of $\left|Z^{L}-Z^{R}\right|$ in Figure 7C, the results are in accordance with the ones in panels A) and B). Namely, at *σ* ^*R*^ = *σ* ^*L*^ = 0.4, which corresponds to the dashed white line, the measure $\left|Z^{L}-Z^{R}\right|$ indicates values close to zero, and the two rings show high coherence. For *σ* ^*R*^ → 1 the two rings present small difference in coherence, independent of the *s*-value. High values of the $\left|Z^{L}-Z^{R}\right|$ measure are recorded at low *σ* ^*R*^ values when *σ* ^*R*^ ≪ *σ* ^*L*^ = 0.4, where the L-ring attains coherence while the L-ring is less coherent due to small coupling strength *σ* ^*R*^ between the nodes.

## 4 Synchronization Patterns for Negative Inter-Ring Coupling

**FIGURE 8:**
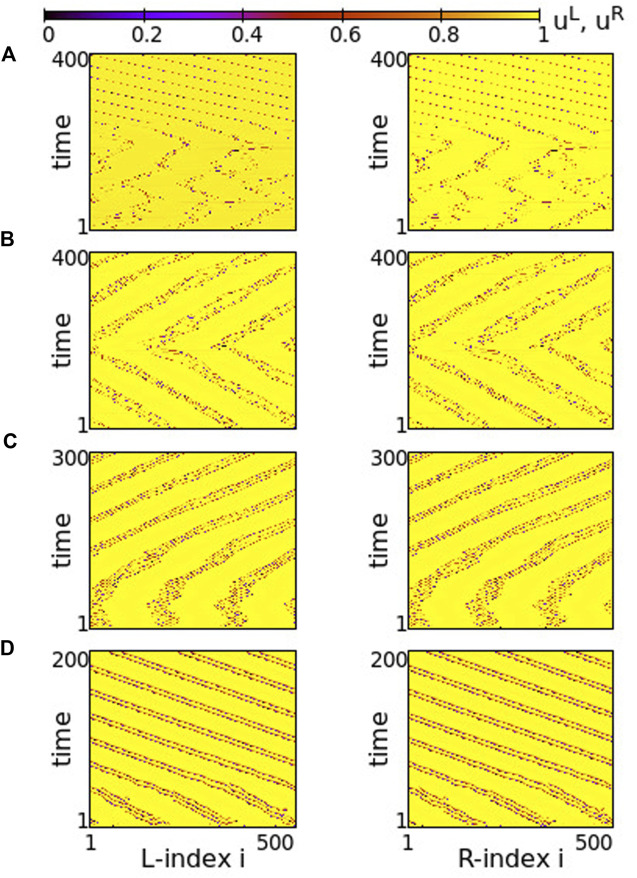
Spacetime plots of the potentials for the L- and R-rings on the left and right panels, respectively. **(A)** *σ* = +1.4, **(B)** *σ* = +1.0, **(C)** *σ* = +0.7 and **(D)** *σ* = +0.5. Parameter *s* = −0.1 and other parameters are as in Figure 2.

Although the inter-ring connectivity is now negative, the main features of synchronization do not change with respect to the previous section. Namely, for positive, large values of the intra-ring coupling strength, most of the elements perform subthreshold fluctuations, while single elements deviate from the subthreshold region and perform full oscillations, see Figure 8A. The full oscillations are not local but travel with constant velocity to the left or to the right, depending on the initial conditions. The motion is in the same direction in both rings.

In Figure 8A, before attaining the above described final state, the elements on each ring organize into four incoherent regions separated by four subthreshold regions. This is transient organization and for long times only single elements escape from the subthreshold potential values. In fact, the duration of the transient organization depends inversely on *σ*. For large positive values of the intra-ring coupling strength, e.g., for *σ* = 2, the transition time tends to zero and the steady state with isolated oscillations travelling around the ring settles immediately (see Supplementary Figure S7). For lower values in this range, such as in Figure 8A, the transition period is finite.

As *σ* decreases in the range 0.8 ≤ *σ* ≤ 1.2, the transient time diverges and the incoherent active regions drift to the left and to the right on each ring for the duration of the simulations (5000 TUs), see Figure 8B. The size and drift velocity of the active regions change randomly with time. We recall that the rest of the elements of the rings do not perform full oscillations, but demonstrate small fluctuations of their potentials just below the threshold *u*_th_.

As the coupling strength decreases further, a certain organization settles in the network. The size and transport velocity of the active regions become constant and for *σ* = +0.7 the size of the active regions does not change with time after transient, see Figure 8C. Decreasing further the *σ* values leads to smaller sizes of the active travelling regions, e.g., for *σ* = +0.5 the travelling active regions consist of only two nodes (see Figure 8D).

Overall comparison between Figures 2, 8 shows that, when the intra-ring coupling takes large or intermediate positive values, similar behaviour is recorded in the two rings, independently of whether the inter-ring values are positive or negative. On the contrary, when the intra-ring couplings are small, positive inter-ring couplings *s* > 0 lead to the formation of well defined, static and wide active regions, while for *s* < 0 the size of the active regions shrinks and their positions change, travelling with constant velocity around the rings.

**FIGURE 9:**
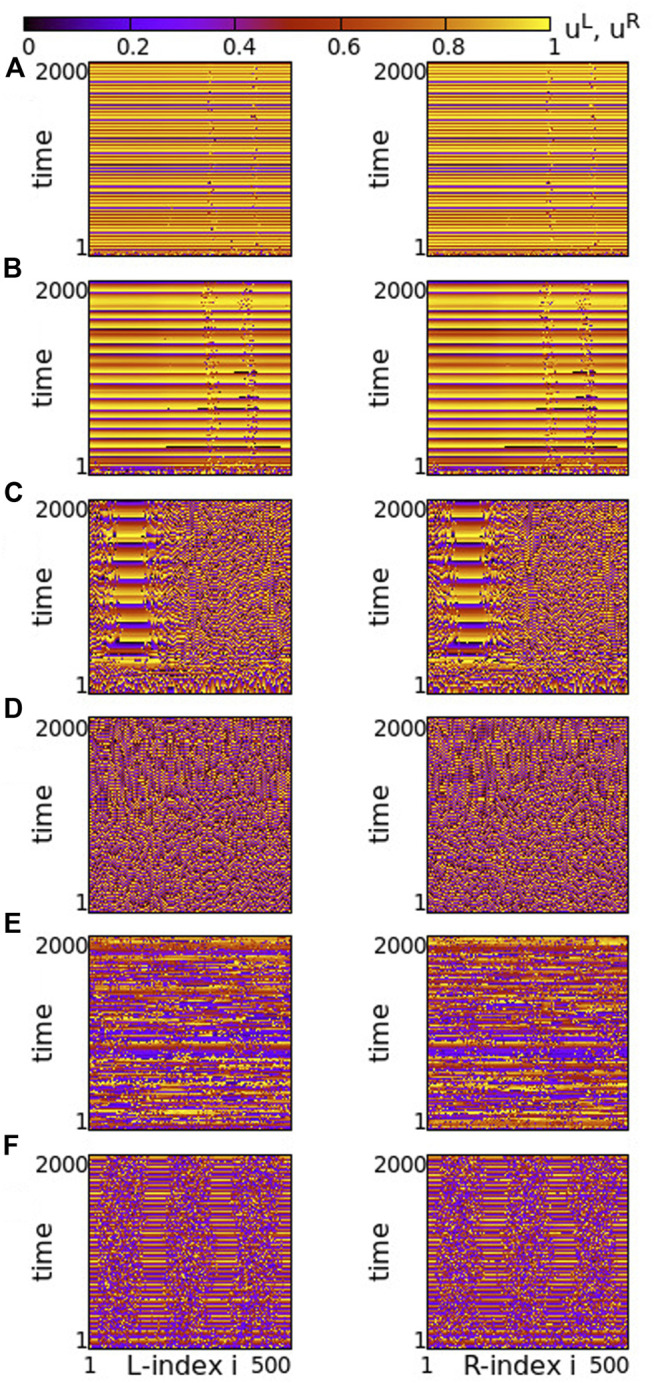
Spacetime plots of the potentials for the L- and R-rings on the left and right panels, respectively. **(A)** *σ* = −0.2, **(B)** *σ* = −0.4, **(C)** *σ* = −0.6, **(D)** *σ* = −0.8, **(E)** *σ* = −1.4 and **(F)** *σ* = −1.9. Parameter *s* = −0.1 and other parameters are as in Figure 2.

Turning now to negative intra-ring couplings, at the same time where *s* = −0.1 inter-ring coupling is considered, we note that the presence of typical chimeras is facilitated. For negative values of *σ* with small modulus, e.g., *σ* = −0.2 in Figure 9A, we note the presence of chimera states with two large coherent and two small incoherent groups, almost identical in both rings. For even smaller moduli (magnitudes) of *σ* the incoherent regions are typical solitary states (see Supplementary Figure S8). As the magnitude of *σ* increases, the size of the two incoherent regions increases in expense of the coherent ones, see Figure 9B. Increasing the absolute value of *σ* further, e.g., for *σ* = −0.6, the size of the incoherent regions in both rings increases. As a result, in each ring the two incoherent regions merge simultaneously forming a large one, bordered by a small coherent region, see Figure 9C. For even larger magnitudes of *σ*, the size of the incoherent region increases further and full incoherence is recorded in both rings of the network, see Figure 9D. As $\left|\sigma \right|>1$ an attempt of organization takes place in both rings as shown in Figure 9E and for very large values of $\left|\sigma \right|$ both rings develop clear chimera states with three coherent and incoherent domains situated at the same positions on the two rings, see Figure 9F.

Overall comparison of Figures 3, 9 shows that for negative intra-ring couplings, when the absolute value $\left|\sigma \right|$ is small, qualitatively we have the formation of solitary states, independently of whether the inter-ring values are positive or negative. In both cases, the system passes from an altogether incoherent state in both rings, while for high values of $\left|\sigma \right|$ chimera states are produced. The chimera states are better pronounced in the case of all (inter- and intra-ring) negative couplings.

**FIGURE 10:**
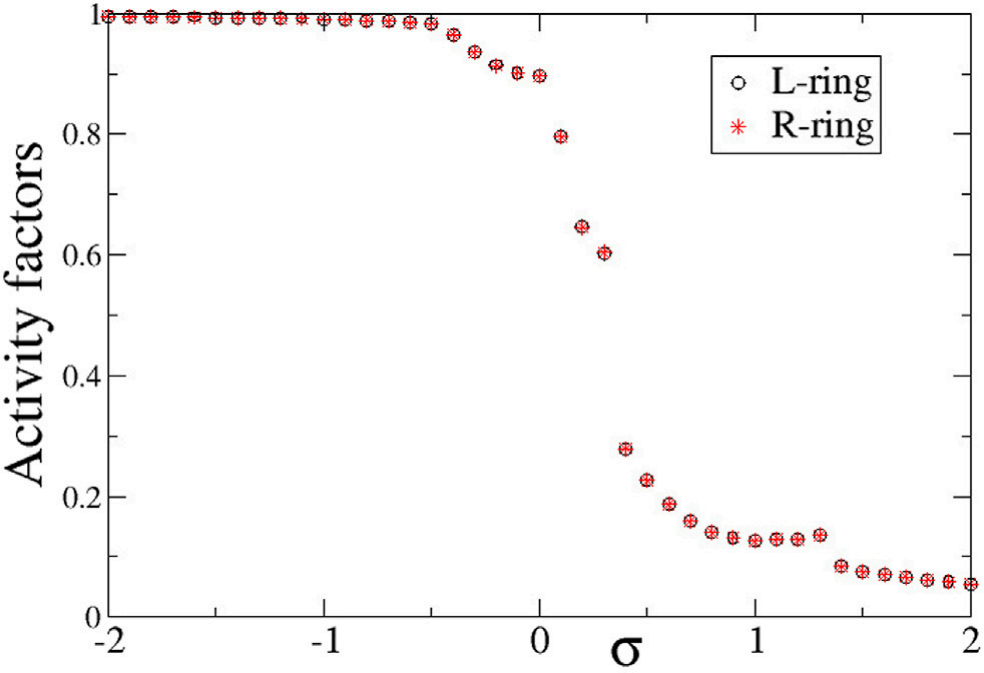
The average activity factors, *A* ^*L*^ (circles), and *A* ^*R*^ (stars) versus the intra-ring coupling strength *σ*. Temporal averages are taken over Δ*T* = 800 TUs, after disregarding the first 200 TUs as transients. Parameter *s* = −0.1 and other parameters are as in Figure 2.

To compare the presence of active and inactive regions in the case of negative inter-ring coupling *s*, we also present in Figure 10 the activity factors *A* ^*L*^ and *A* ^*R*^ as a function of the common intra-ring coupling *σ*. Similarly, to the case of *s* > 0 described in the previous section, here also the activity is extended over all elements for negative *σ*, while it is restricted for *σ* > 0 due to the presence of the subthreshold elements. For *σ* > 0, the activity reduces inversely with the intra-ring coupling strength following the same scenario as for the case of *s* < 0 (see Section 3).

**FIGURE 11:**
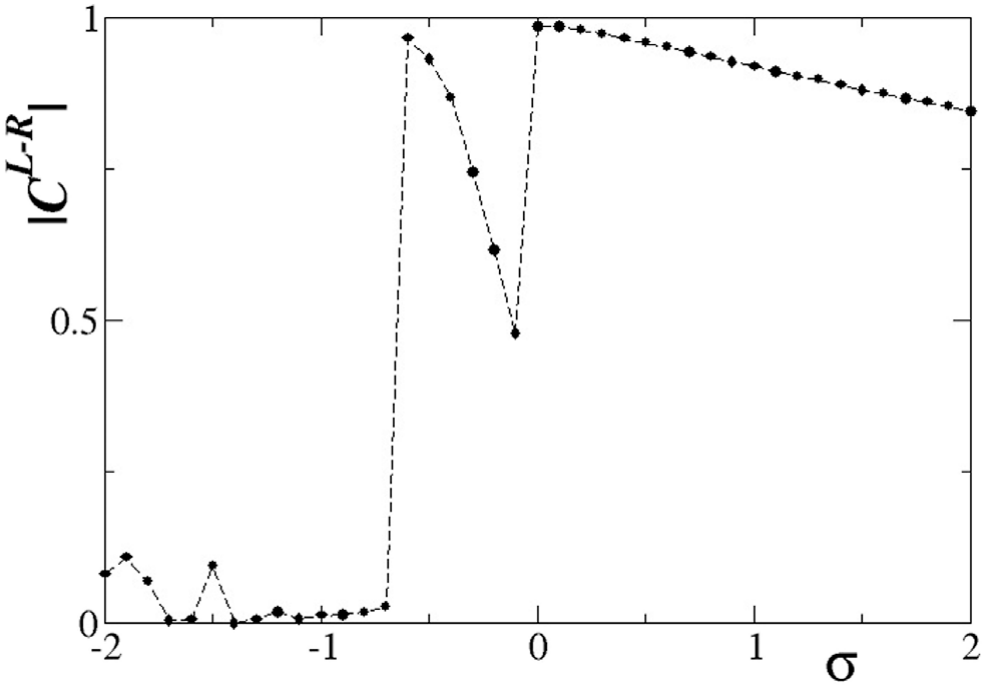
The correlation function, $\left|C^{L-R}\right|$ , versus the intra-ring coupling strength *σ*. Parameter *s* = −0.1 and other parameters are as in Figure 2.

Quantitative results on the L-R correlation function for *s* = −0.1 are presented in Figure 11. Starting with large, positive values of the intra-ring coupling *σ*, we note a high degree of correlation between the two rings. This can be confirmed from Figure 8, where the subthreshold and active regions appear in identical locations on the two rings. At *σ* = 0 the correlations decrease but do not drop to zero, since the inter-ring coupling *s* ≠ 0 imposes a certain synchrony between L- and R-rings. For inhibitory *σ* values with small amplitudes, solitary states are developed in both rings at the same positions, and we note from Figure 9 that the coherent domains have the same phase in the L- and R-rings. This causes increased values of the correlation function as confirmed in Figure 11 for −0.6 < *σ* < 0. For intermediate negative intra-ring coupling values, − 1.6 < *σ* < − 0.6, the system demonstrates full incoherence in both rings, before giving rise to chimera states which appear for −2.0 < *σ* < − 1.6, as shown in Figures 9C–F. These behaviours are mirrored in the correlation function, Figure 11: for $-1.6<\sigma <-0.6\;\left|C^{L-R}\right|$ drops to zero, while for −2.0 < *σ* < − 1.6 the coherent and incoherent parts of the chimera states appear in identical positions on the two rings and this leads to a certain, finite degree of correlations.

**FIGURE 12:**
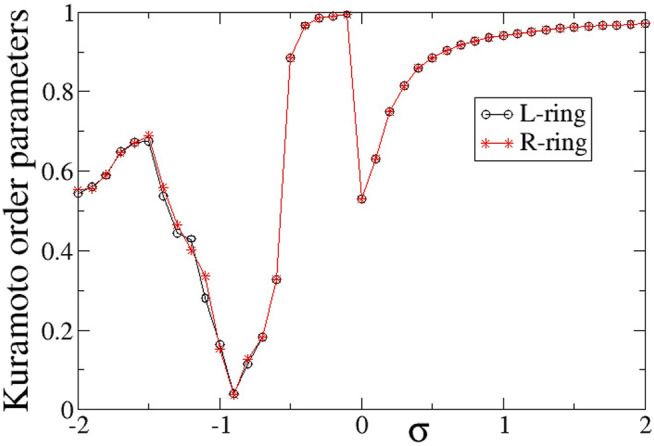
The Kuramoto order parameters, *Z* ^*L*^ (circles) and *Z* ^*R*^ (stars), versus the intra-ring coupling strength *σ*, for negative inter-ring coupling *s* and equal intra-ring couplings *σ* ^*L*^ = *σ* ^*R*^ = *σ*. Parameter *s* = −0.1 and other parameters are as in Figure 2.

To quantify the degree of synchronization within each ring, the Kuramoto order parameters *Z* ^*L*^ (circles) and *Z* ^*R*^ (stars) are plotted in Figure 12 as a function of the intra-ring coupling range *σ* ^*L*^ = *σ* ^*R*^ = *σ* for negative inter-ring coupling *s*. The *Z*-curve in both rings is almost identical to each other and very similar to the case of *s* > 0, shown in Figure 6. Namely, for *σ* < − 0.5 the rings are in the chimera realm with *Z* ^*L*^ ≃ *Z* ^*R*^ < 1, for −0.5 < *σ* < 0 the rings are characterised by rare solitaries with *Z* ^*L*^ ≃ *Z* ^*R*^ → 1, for 0 < *σ* < 1 both rings are composed by alternation of subthreshold and active regions with *Z* ^*L*^ ≃ *Z* ^*R*^ < 1 and for *σ* > 1 the size of the active regions tend to zero reducing to single active elements travelling around the rings and the *Z* order parameters tend to 1 as *σ* increases above unity. As in the case of inter-ring coupling *s* > 0, the two transitions (qualitative change of behaviour) between chimeras and solitaries (at *σ* ∼ − 0.5) and between solitary states and subthreshold/active regions (at *σ* ∼ 0) are abrupt reminding some phase transitions in physical systems.

**FIGURE 13:**
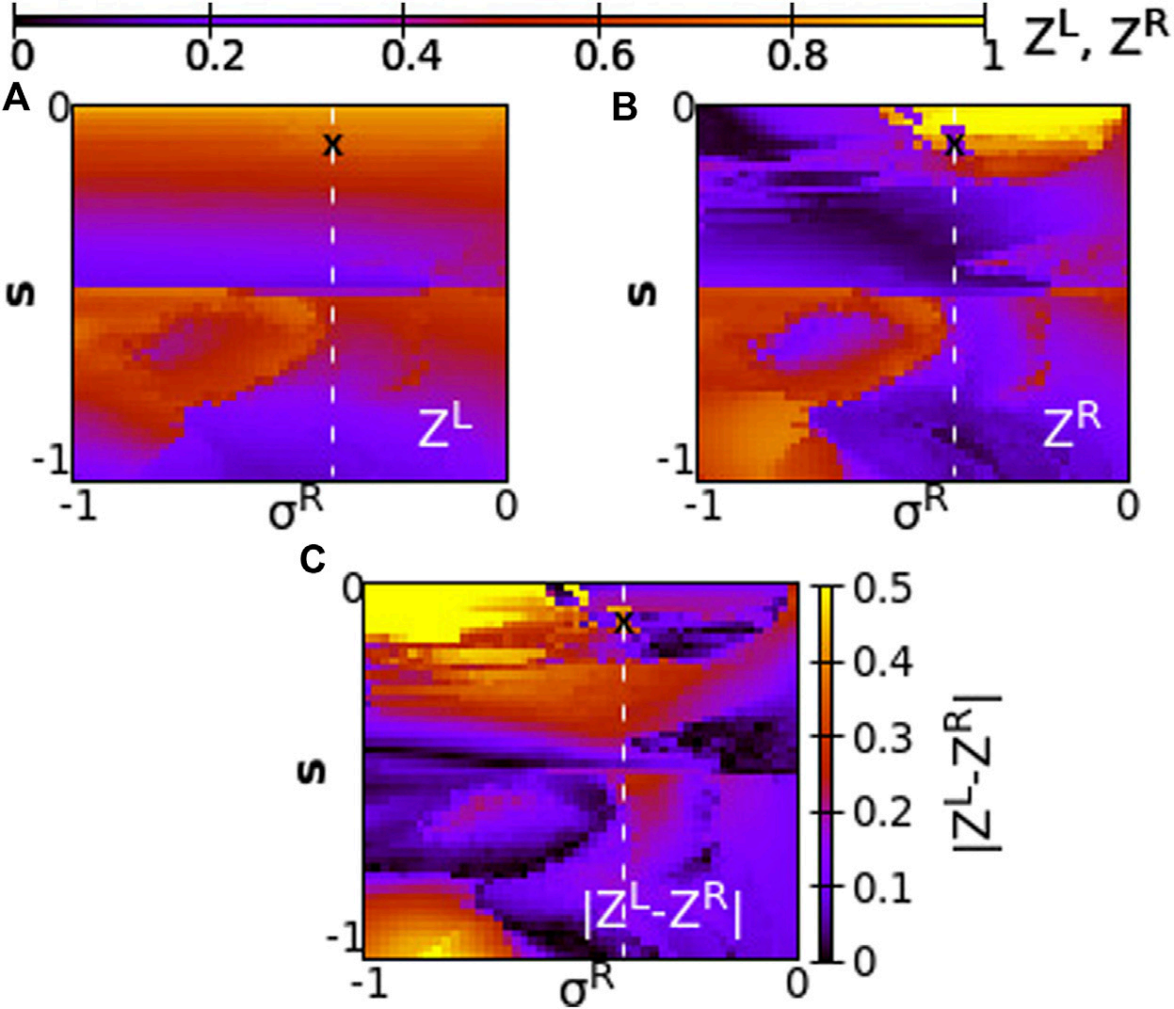
The Kuramoto order parameters *Z* ^*L*^ and *Z* ^*R*^ represented on the color scale for different values of the intra-ring coupling constant in R-ring, *σ* ^*R*^, and the inter-ring coupling constant, *s*. **(A)** *Z* ^*L*^, **(B)** *Z* ^*R*^ and **(C)** $\left|Z^{L}-Z^{R}\right|$ . The white dashed line represents equal intra-ring couplings *σ* ^*L*^ = *σ* ^*R*^ = −0.4 and variable $s\in \left[-1,0\right]$ . The “X” symbol marks coupling strengths *σ* ^*L*^ = *σ* ^*R*^ = −0.4 and *s* = −0.1, discussed in Figure 9B. Parameter *σ* ^*L*^ = −0.4 and other parameters are as in Figure 2.

Following a similar reasoning as in Section 3, we study the network synchrony when the two rings have different coupling strengths, *σ* ^*L*^ ≠ *σ* ^*R*^ < 0. To this purpose, we perform numerical simulations of the network keeping constant *σ* ^*L*^ = −0.4 and we vary *σ* ^*R*^ and the inter-ring coupling *s* in the range $\left[-1,0\right]$ . This way all couplings are negative in both rings. To quantify the synchrony in the system, we present in Figure 13 the maps of the Kuramoto order parameters. For these parameter values, chimera states are recorded and the results refer to the images in Figure 9, where all coupling strengths are also negative. Following the notation of the previous section, in all Kuramoto maps the coupling strength *σ* ^*R*^ varies along the *x*-axis, the inter-ring coupling *s* varies along the *y*-axis and *σ* ^*L*^ = −0.4 remains constant in all simulations. The *Z* values for *σ* ^*L*^ = *σ* ^*R*^ = −0.4 and *s* = −0.1 are marked by the symbol “X” in Figure 13 and correspond precisely to the state represented in Figure 9B.

As Figure 13 demonstrates, for negative coupling strengths the variations of the Kuramoto order parameter show a more chaotic pattern than for positive strengths, owing to the creation and destruction of chimera states. In many cases low *Z* values are recorded in both rings indicating a low degree of coherence, as for *σ* ^*R*^ ≃ − 1 and −0.5 < *s* < − 0.4. For other coupling values, e.g., *σ* ^*R*^ ≃ − 1 and −0.8 < *s* < − 0.5 high values of *Z* are recorded in both rings indicating either chimera states with large, synchronous coherent domains or solitary states with synchronous coherent regions.

Regarding the values of $\left|Z^{L}-Z^{R}\right|$ in Figure 13C, the black colors indicate that the two rings operate in coherence for these parameter regions, because the terms corresponding to a certain oscillator *i* in the two rings cancel out. Alternatively, the red-yellow regions show decoherence between the two rings. Decoherence is mostly visible for small values of *s*, where communication is minimal between the two rings and also when *σ* ^*R*^ → 0 while *σ* ^*L*^ keeps a finite value equal to -0.4. In particular, for the parameters marked by “X” in the plot, *σ* ^*L*^ = *σ* ^*R*^ = −0.4 and *s* = −0.1, both rings have similar *Z* values which cancel out and therefore, the corresponding point in panel C) has dark color.

Interesting parameter regions from the point of view of brain-related functions are the ones that allow different types of coherence in the two rings, since during brain activity one hemisphere may operate independently of the other as testified by different synchronization patterns. A related discussion follows in the next section, where additional simulations are presented using smaller inter-ring coupling values, *s* = +0.01 and *s* = −0.01.

## 5 Weak Multiplexing

In this final section, we include some intriguing results for very small values of inter-ring couplings, namely *s* = +0.01 and *s* = −0.01. Small coupling strengths prevail in the connectivity between the two hemispheres of the brain, as compared to the intra-hemisphere couplings and therefore, the study of weak inter-ring connectivity in multiplex networks can be of interest for medical brain applications. We only present here the cases which deviate from the above discussion. In particular, we note two cases where qualitatively different behaviour is reported in the L- and R-ring.

In Section 4 we noted that, as we increase (or decrease) the parameter *σ*, we have a qualitative transition in the behaviour from one type of synchronization to another, e.g., transitions from both rings being fully asynchronous to chimera states, as in Figure 9. Due to the finite size of the inter-ring coupling, each ring influences the other and they both attain the same long time state. When the inter-ring coupling takes small values, e.g., $\left|\sigma \right|=0.01$ , then the influence between rings is weak and different synchronization patterns may appear in the two rings.

**FIGURE 14:**
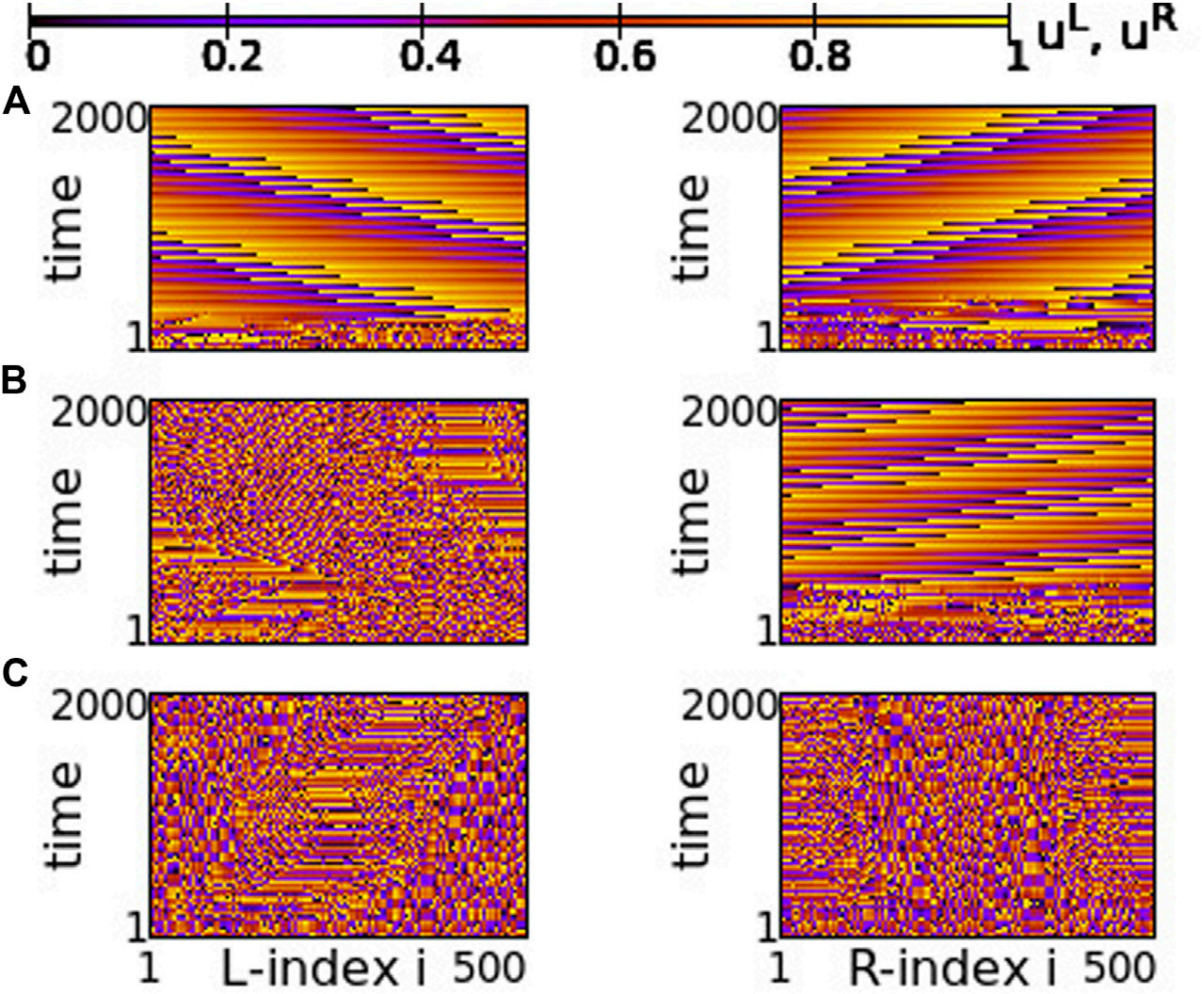
Spacetime plots of the potentials for the L- and R-rings on the left and right panels, respectively. **(A)** *σ* = −0.6, **(B)** *σ* = −0.7, and **(C)** *σ* = −0.8. Parameter *s* = +0.01 and other parameters are as in Figure 2.

The case of small positive *s* = +0.01 is plotted in Figure 14. For negative values of *σ* ≥ −0.6 both rings synchronize and travelling waves are recorded at long times, see Figure 14A. For *σ* ≤ −0.8 chimera states with one synchronous and one asynchronous region are presented in both rings, see Figure 14C. Precisely at the critical point of parameter *σ* where the behaviour changes qualitatively, namely at *σ* = −0.7, one ring supports a chimera state while the other develops travelling waves, see Figure 14B. This takes place only in rings of finite size (here *N* ^*L*^ = *N* ^*R*^ = 500), where the initial conditions favour one or the other behaviour, when the coupling is not strong enough to impose one, common synchronization pattern (either chimera state or travelling waves in both rings).

**FIGURE 15:**
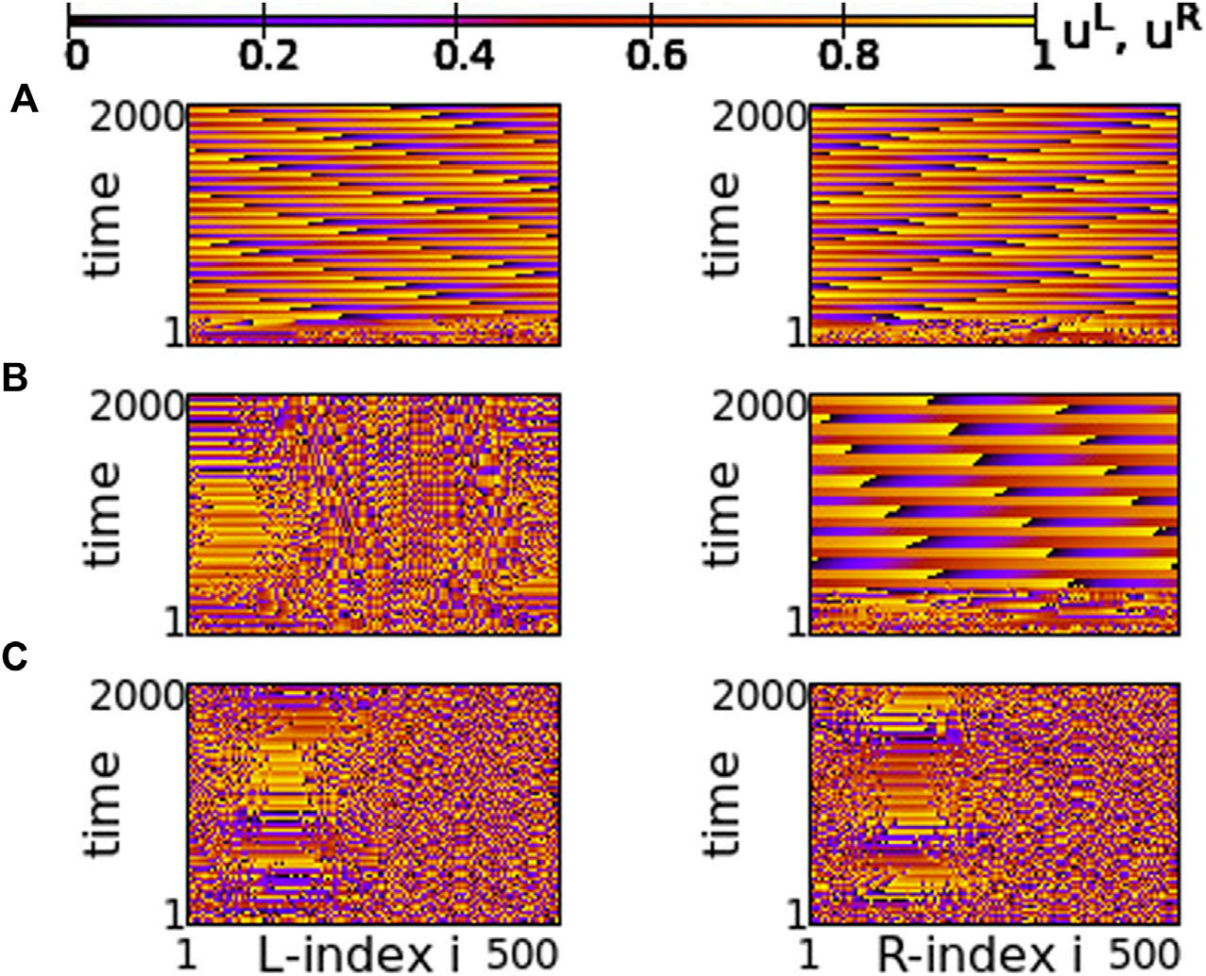
Spacetime plots of the potentials for the L- and R-rings on the left and right panels, respectively. **(A)** *σ* = −0.5, **(B)** *σ* = −0.6, and **(C)** *σ* = −0.7. Parameter *s* = −0.01 and other parameters are as in Figure 2.

A similar situation is also reported for *s* = −0.01 and *σ* = −0.6, in Figure 15. For this value of the inter-coupling strength, *s* = −0.01, the transition between chimera patterns and travelling fronts takes place at a different value of *σ* = −0.6. For *σ* > − 0.6 travelling fronts are recorded in both rings, see Figure 15A and for *σ* < − 0.6 chimera states develop, see Figure 14C. At the critical point between the two regimes, *σ* = −0.6, one of the rings develops the chimera state, while travelling fronts are developed in the other, similarly to the case of *s* = +0.01. Note that here the chimera motifs (for *σ* = −0.6 and -0.7) are better defined and localized, since all coupling strengths in the system are negative.

## 6 Conclusion and Open Problems

Motivated by the division of the brain into two distinct hemispheres with intra- and inter-connections between them, we study here the synchronization properties of a multiplex network consisting of two semirings with nonlocal connectivity between the nodes in each ring and with one-to-one connectivity across rings. The nodes of the network are modelled as LIF oscillators, while the connectivity values take both positive and negative values, indicating excitatory and inhibitory connections, respectively. Using the Kuramoto order parameter as an index of synchronization within each ring and the correlation function as an indicator of inter-ring synchrony, we explore the parameter regions where different synchronization patterns prevail. Typical such patterns range from full synchrony in both rings, to solitary states, chimera states and full incoherence. The interesting phenomenon of coexistence of different patterns in the two rings for the case of weak multiplexing is also reported.

In the present study the inter-ring connectivity is taken as a one-to-one linking. However, biological evidence indicates that the local regions in one hemisphere may be connected with multiple centers in the opposite hemisphere (Finn et al., 2015). A step in this direction would be to consider reflecting connectivity in the multiplex, where each node of the R-ring is coupled to many nodes in the opposite ring in addition to the intra-ring links (and similarly for the nodes of ring L).

Since it is hard to obtain the precise connectivity in most real-world networks, many studies of coupled oscillators on simplex and multiplex networks avoid to use deterministic nonlocal connectivity but introduce stochasticity in the network, such as addition of random long distance links (small-world connectivity) or noise in the coupling strengths (Omelchenko et al., 2015a; Argyropoulos and Provata, 2019; Olmi et al., 2019). It would be interesting to test the effects of stochasticity on the LIF multiplex network, by introducing randomness on the inter- and/or intra-ring couplings.

In multiplex connectivity, it is often the case that one may influence the dynamics of one ring by performing modifications in the other (Ruzzene et al., 2020). This is a useful procedure when one of the rings is not accessible to the user. It is interesting to investigate this remote type of synchronization in the LIF multiplex network, addressing questions such as, is it possible to modify synchronization patterns in the L-ring by performing connectivity changes in the R-ring, or is it possible to drive the L-ring to full synchrony by applying a pacemaker with specific frequency to the R-ring?

A last open problem relates to the dynamics of the single LIF oscillator. As discussed in Section 2, biological neurons are characterized by a refractory period. This is the period that the neuron remains at the rest state after resetting and corresponds roughly to half a period of the single neuron. The addition of a refractory period in simple ring networks composed of LIF elements causes transitions from single to multichimera states. It would be interesting to investigate the influence of the refractory period in multiplex networks and the corresponding synchronization phenomena.

## Data Availability

The raw data supporting the conclusion of this article will be made available by the authors, without undue reservation.
