# Supplementary figures and images for: Synchronization in Multiplex Leaky Integrate-and-Fire Networks With Nonlocal Interactions
Source: Front Netw Physiol. 2022 Jun 29;2:910862. doi: 10.3389/fnetp.2022.910862 (PMC10013047; doi:10.3389/fnetp.2022.910862)

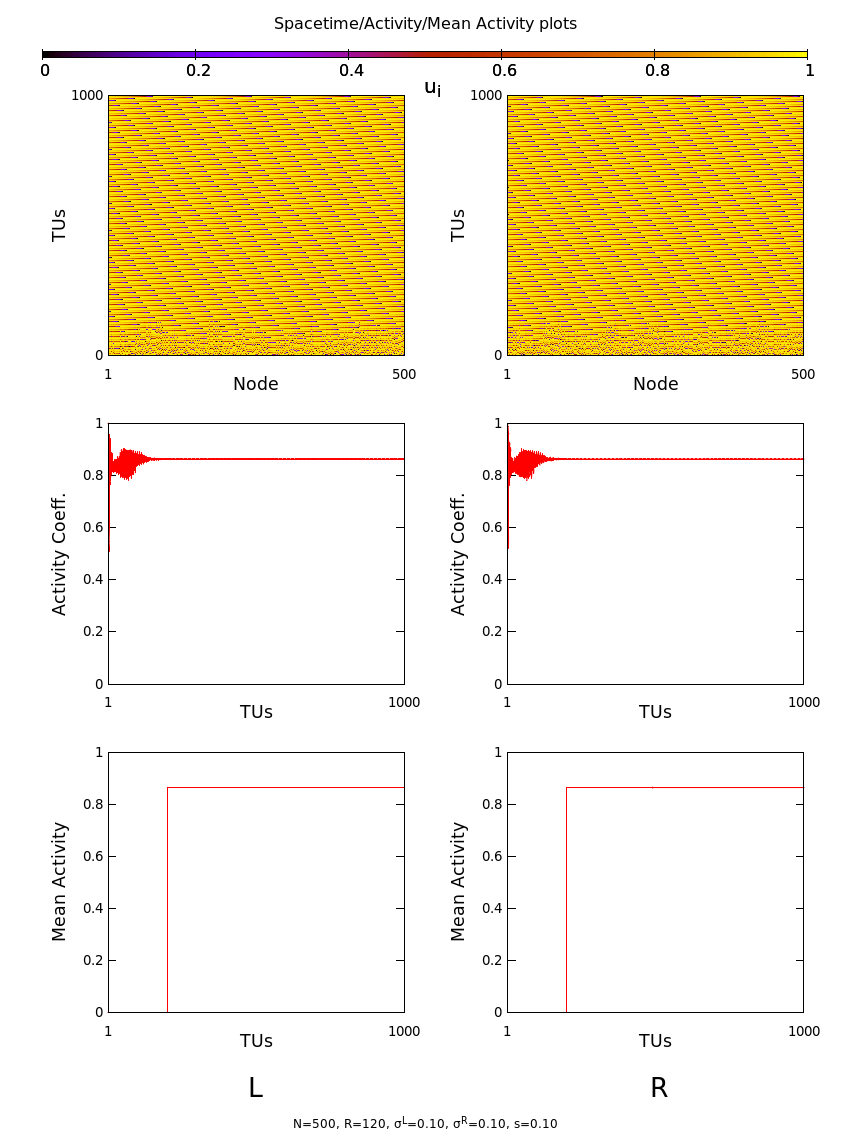

Supplement: Supplementary file 1 [file Image11.PNG]

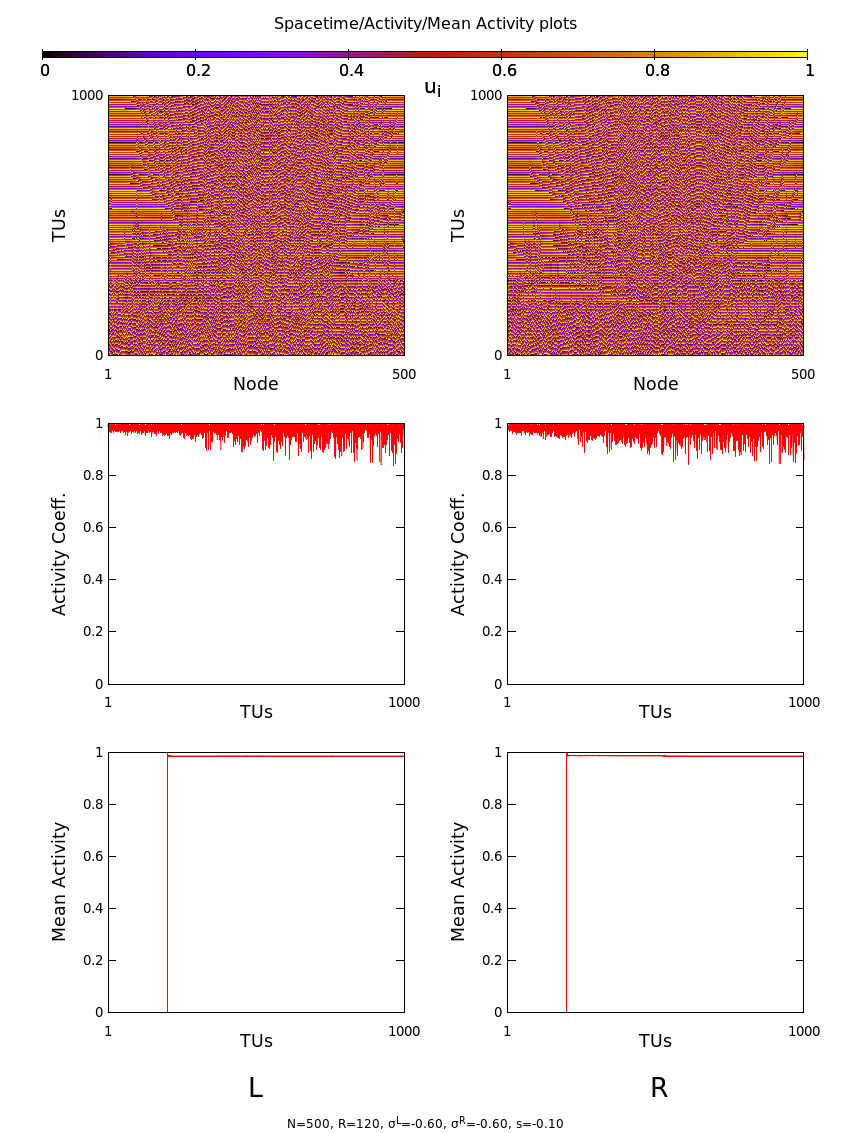

Supplement: Supplementary file 2 [file Image12.PNG]

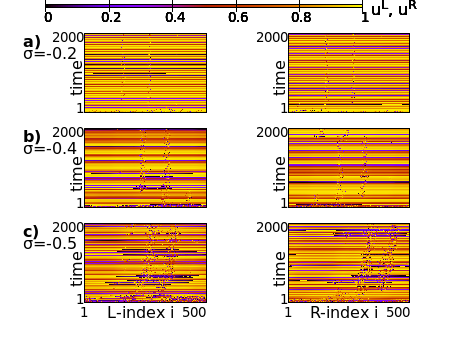

Supplement: Supplementary file 3 [file Image5.PNG]

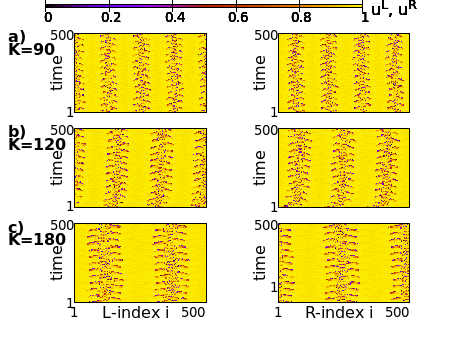

Supplement: Supplementary file 4 [file Image4.PNG]

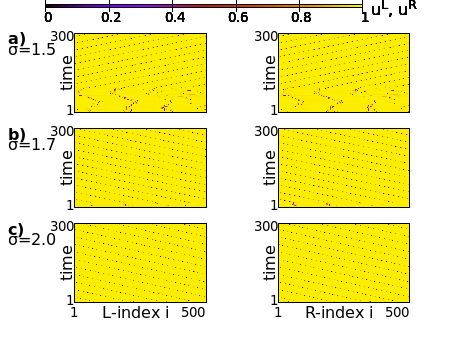

Supplement: Supplementary file 6 [file Image7.PNG]

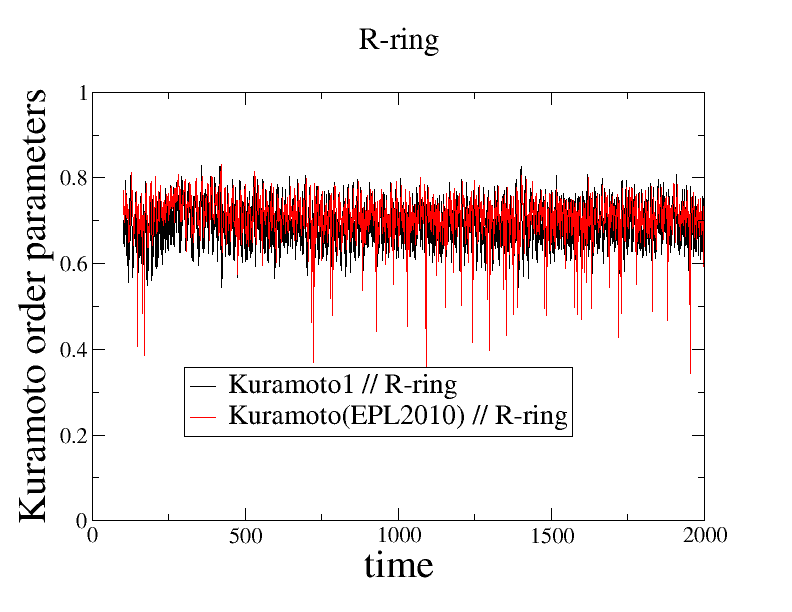

Supplement: Supplementary file 7 [file Image2.PNG]

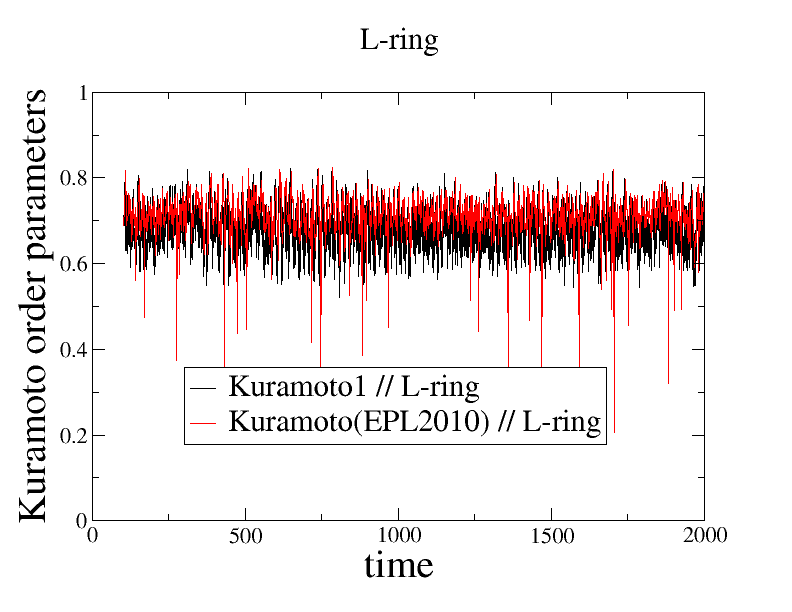

Supplement: Supplementary file 8 [file Image1.PNG]

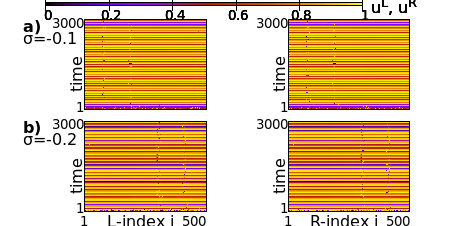

Supplement: Supplementary file 9 [file Image8.PNG]

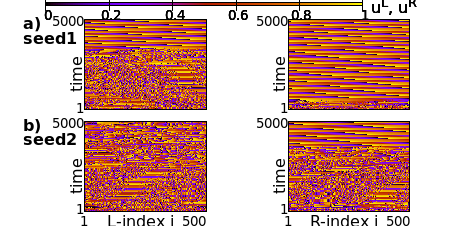

Supplement: Supplementary file 10 [file Image9.PNG]

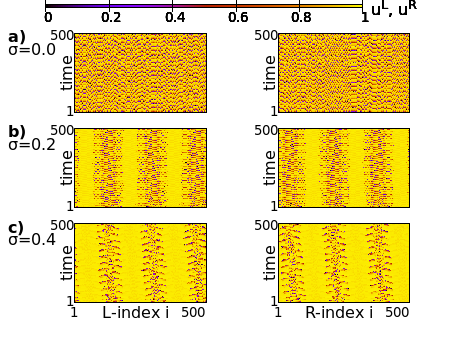

Supplement: Supplementary file 11 [file Image6.PNG]

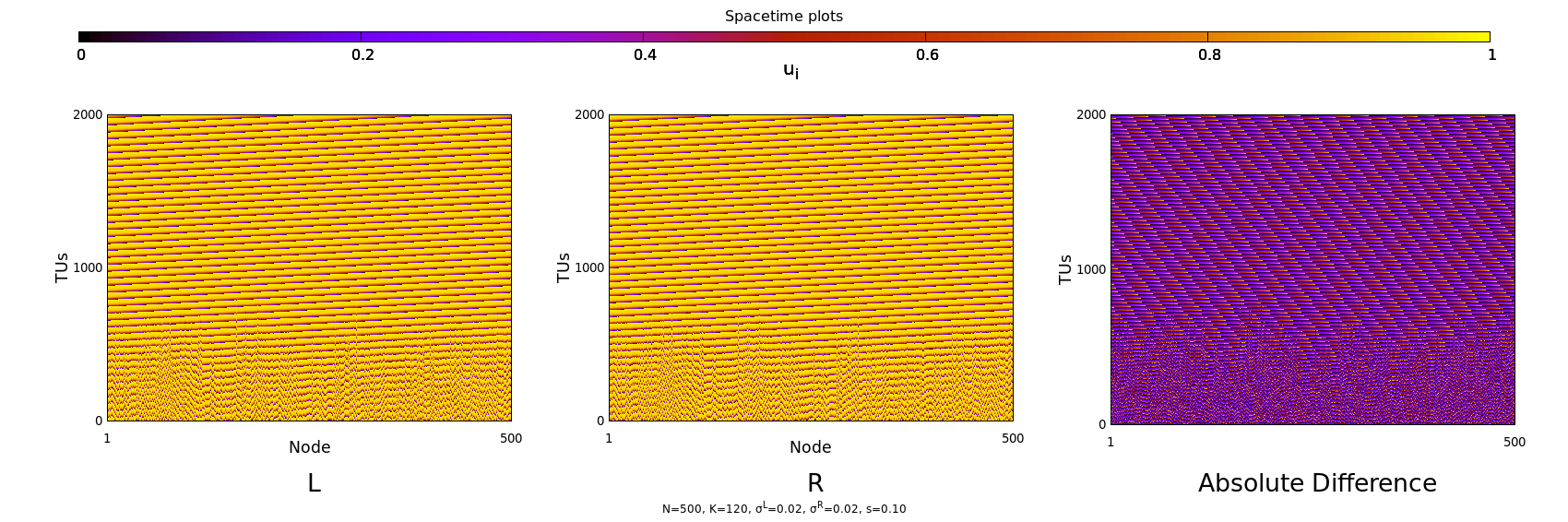

Supplement: Supplementary file 12 [file Image3.PNG]

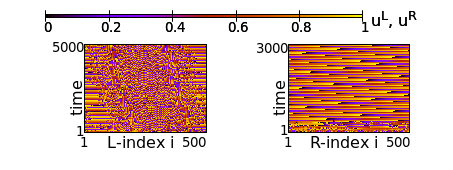

Supplement: Supplementary file 13 [file Image10.PNG]
