# Supplementary material for: Synchronization in Multiplex Leaky Integrate-and-Fire Networks With Nonlocal Interactions
Source: Front Netw Physiol. 2022 Jun 29;2:910862. doi: 10.3389/fnetp.2022.910862 (PMC10013047; doi:10.3389/fnetp.2022.910862)
Supplement: Supplementary file 5 [file DataSheet1.PDF]

Descriptions of Supplementary Figures

Fig-supplement-01.png

Calculation of the Kuramoto order parameter using two different phase definitions. By "Kuramoto(EPL2010)" (**red curves**) we denote the results using the definition proposed in Ref. [7 previous version]=Ref. [8 current version] and by "Kuramoto1" (**black curves**) the definition in the present study and we plot them as a function of time for 2000TUs.
In this figure only the results on the L-ring are shown. We find that both phase definitions give compatible results. Parameter values are s=0.1, $\sigma^L = \sigma^R = -1.5$ and other parameters as in Fig. 2 of the manuscript, which produce a chimera state (with three coherent regions).

Fig-supplement-02.png

Same as previous (Fig-supplement-01.png) but the results are for the R-ring.

Fig-supplement-03.png (lines 471-472):

Exemplary spacetime plots of the potentials of left ring (left panel), right ring (middle panel) and absolute potential difference between left and right rings (right panel), when the intra-ring coupling strengths on the left and right rings are small. Here σ^L=σ^R=0.02. We note that the steady states in both rings are coherent traveling waves of the same form. Other parameters are: $N^L = N^R = N = 500$, s = +0.1, K = 120, μ = 1, $u_{rest} = 0$, u_{th} = 0.98.

Fig-supplement-04.png (line 486- 487):

Exemplary spacetime plots of the potentials of left ring (left panel), right ring (right panel) for different values of the coupling range K. We note that the number of active and subthreshold regions decreases inversely with K. For K=90 we note 4 active/subthreshold regions, for K=120 we note 3 and for K=180 we note 2 active/subthreshold regions. Other parameters are: $N^L = N^R = N = 500$, σ^L=σ^R=0.4, s = +0.1, μ = 1, $u_{rest} = 0$, u_{th} = 0.98.

Fig-supplement-05.png (line 509-510):

Exemplary spacetime plots of the potentials of left ring (left panel), right ring (right panel) for different values of negative coupling strangths. When the coupling strengths are negative and have small absolute values solitary states are formed. As σ^L and σ^R increase in absolute values the size of the incoherent regions increases. We present here the cases of a) σ^L=σ^R= -0.2 , b) σ^L=σ^R= -0.4 and c) σ^L=σ^R= -0.5. Other parameters are: $N^L = N^R = N = 500$, s = +0.01, K = 120, μ = 1, $u_{rest} = 0$, u_{th} = 0.98.

Fig-supplement-06 .png (line 575-576):

Exemplary spacetime plots of the potentials of left ring (left panel), right ring (right panel) for different values of zero and small positive coupling strengths, paying attention to similarity between left and right rings. When the coupling strengths are zero a certain degree of correlation (similarity) develops between the L- and R rings, due to the positive

intrer-ring coupling strength s. For positive coupling strengths $\sigma^L=\sigma^R>0$, the correlations drop with the intra-ring coupling strength, due mostly to the opposite locations of the active regions in the two rings. We present here the cases of a) $\sigma^L=\sigma^R= 0.0$, b) $\sigma^L=\sigma^R= 0.2$ and c) $\sigma^L=\sigma^R= 0.4$. Other parameters are: $N^L = N^R = N = 500$, s = 0.1, K = 120, μ = 1, $u_{rest} = 0$, $u_{th} = 0.98$.

Fig-supplement-07.png (line 698-699):

Exemplary spacetime plots of the potentials of left ring (left panel), right ring (right panel) for different values of positive coupling strengths, paying attention to the setting time of the steady state. When the coupling strengths are positive, the larger the coupling σ the earlier the steady state (traveling fronts) settles. We present here the cases of a) $\sigma^L=\sigma^R= 1.5$, where the steady state settles at about 100 TU, b) $\sigma^L=\sigma^R= 1.7$ where the steady state settles at about 10 TU and c) $\sigma^L=\sigma^R= 2.0$ where the steady state settles almost immediately. Other parameters are: $N^L = N^R = N = 500$, s = -0.1, K = 120, μ = 1, $u_{rest} = 0$, $u_{th} = 0.98$.

Fig-supplement-08.png (line 737-738):

Exemplary spacetime plots of the potentials of left ring (left panel), right ring (right panel) for different values of negative coupling strengths, paying attention the development of solitary states. When the coupling strengths are negative with small modulus, solitary states appear in both rings. We present here the cases of a) $\sigma^L=\sigma^R= -0.1$ and b) $\sigma^L=\sigma^R= -0.2$ where solitaries tend to mobilize to form two incoherent domains in each ring. Other parameters are: $N^L = N^R = N = 500$, s = -0.1, K = 120, μ = 1, $u_{rest} = 0$, $u_{th} = 0.98$.

Fig-supplement-09.png

Exemplary spacetime plots of the potentials of left ring (left panel), right ring (right panel) for different random initial conditions/potentials (realized by seed1 and seed2), for the case of weak multiplexing. **First row:** For this initial conditions (seed1)**,** the two rings acquire qualitatively different steady states up to time 3500 TU, but after that time the L-ring changes to traveling waves following the state of the R-ring. **Second row:** For this initial conditions (seed2)**,** the two rings keep qualitatively different steady states up to time 5000 TU, at least. Parameters are: $N^L = N^R = N = 500$, $\sigma^L=\sigma^R= -0.6$, **s = 0.01 (weak multiplexing)**, K = 120, μ = 1, $u_{rest} = 0$, $u_{th} = 0.98$.

Fig-supplement-10.png

Extending the time axis up to 5000TUs in Figure 15b (numbering in current version). The two rings keep qualitatively different steady states up to time 5000 TU, at least. Parameters are: $N^L = N^R = N = 500$, $\sigma^L=\sigma^R= -0.6$, **s = 0.01 (weak multiplexing)**, K = 120, μ = 1, $u_{rest} = 0$, $u_{th} = 0.98$.

Fig-supplement-11.png

On the calculations of the activity factor $A^L(t)$ and $A^R(t)$, using Eq. (9), for positive

coupling strengths (values $\sigma^L = \sigma^R = 0.1$, $s=0.1$) and other parameters are as in Fig. 2 of the manuscript.

- Top row: Spacetime plots of the potentials in the L- and R- rings.
- Middle row: Instantaneous activity factor with time in the l L- and R- rings.
- Last row: Temporal average of the activity factor in the L- and R- rings.
  For the temporal average activities (last row) the first 200TUs were disregarded as transients.

<u>Fig-supplement-12.png</u>

On the calculations of the activity factor $A^L(t)$ and $A^R(t)$, using Eq. (9), for negative coupling strengths (values $\sigma^L = \sigma^R = -0.6$, $s=-0.1$) and other parameters are as in Fig. 2 of the manuscript.

- Top row: Spacetime plots of the potentials in the L- and R- rings.
- Middle row: Instantaneous activity factor with time in the l L- and R- rings.
- Last row: Temporal average of the activity factor in the L- and R- rings.
  For the temporal average activities (last row) the first 200TUs were disregarded as transients.
